# Patterns of brain dynamic functional connectivity are linked with attention-deficit/hyperactivity disorder-related behavioral and cognitive dimensions

**DOI:** 10.1017/S0033291723000089

**Published:** 2023-10

**Authors:** Lekai Luo, Lizhou Chen, Yuxia Wang, Qian Li, Ning He, Yuanyuan Li, Wanfang You, Yaxuan Wang, Fenghua Long, Lanting Guo, Kui Luo, John A. Sweeney, Qiyong Gong, Fei Li

**Affiliations:** 1 Huaxi MR Research Center (HMRRC), Department of Radiology, West China Hospital of Sichuan University, Chengdu 610041, Sichuan, P.R. China; 2 Research Unit of Psychoradiology, Chinese Academy of Medical Sciences, Chengdu 610041, Sichuan, P.R. China; 3 Functional and Molecular Imaging Key Laboratory of Sichuan University, Chengdu 610041, Sichuan, P.R. China; 4 Department of Psychiatry, West China Hospital of Sichuan University, Chengdu 610041, Sichuan, P.R. China; 5 National Engineering Research Center for Biomaterials, Sichuan University, Chengdu 610041, Sichuan, P.R. China; 6 Department of Psychiatry and Behavioral Neuroscience, University of Cincinnati, Cincinnati, OH 45219, USA; 7 Department of Radiology, West China Xiamen Hospital of Sichuan University, Xiamen 361021, Fujian, P.R China

**Keywords:** Attention-deficit/hyperactivity disorder, cognitive function, dynamic functional connectivity, resting-state functional MRI, sparse canonical correlation analysis

## Abstract

**Background:**

Attention-deficit/hyperactivity disorder (ADHD) is a clinically heterogeneous neurodevelopmental disorder defined by characteristic behavioral and cognitive features. Abnormal brain dynamic functional connectivity (dFC) has been associated with the disorder. The full spectrum of ADHD-related variation of brain dynamics and its association with behavioral and cognitive features remain to be established.

**Methods:**

We sought to identify patterns of brain dynamics linked to specific behavioral and cognitive dimensions using sparse canonical correlation analysis across a cohort of children with and without ADHD (122 children in total, 63 with ADHD). Then, using mediation analysis, we tested the hypothesis that cognitive deficits mediate the relationship between brain dynamics and ADHD-associated behaviors.

**Results:**

We identified four distinct patterns of dFC, each corresponding to a specific dimension of behavioral or cognitive function (*r* = 0.811–0.879). Specifically, the inattention/hyperactivity dimension was positively associated with dFC within the default mode network (DMN) and negatively associated with dFC between DMN and the sensorimotor network (SMN); the somatization dimension was positively associated with dFC within DMN and SMN; the inhibition and flexibility dimension and fluency and memory dimensions were both positively associated with dFC within DMN and between DMN and SMN, and negatively associated with dFC between DMN and the fronto-parietal network. Furthermore, we observed that cognitive functions of inhibition and flexibility mediated the relationship between brain dynamics and behavioral manifestations of inattention and hyperactivity.

**Conclusions:**

These findings document the importance of distinct patterns of dynamic functional brain activity for different cardinal behavioral and cognitive features related to ADHD.

## Introduction

Attention-deficit/hyperactivity disorder (ADHD) is a heterogeneous condition manifest in problems of inattention, hyperactivity, impulsivity, and deficits in cognitive function (Willcutt, Doyle, Nigg, Faraone, & Pennington, 2005). These problems are believed to be driven by atypical brain structural (Aoki, Cortese, & Castellanos, 2018) and functional network organization (Cortese et al., 2016). Abnormal functional connectivity (FC) within and between default-mode, cognitive control, and attention networks (Castellanos & Aoki, 2016; Li et al., 2014) has been identified in individuals with ADHD. Most studies have traditionally investigated static connectivity in case–control comparisons, which assume a stable homogenous pattern of FC over time. Both empirical observations and emerging theoretical perspectives indicate that the brain connectome is time-varying and dynamic in ways that are themselves clinically relevant. Thus, measuring brain dynamic fluctuations may provide unique insight into dysfunctional brain organization and its relation to specific behavioral issues in ADHD (Rolls, Cheng, & Feng, 2021). Most previous studies of dynamic FC (dFC) in ADHD rely on case–control study designs, testing for categorical differences between patients and controls. These studies reported greater variability in brain FC over time at the global brain level (de Lacy & Calhoun, 2019), locally within default mode network (DMN) (Mowinckel et al., 2017) or between DMN and other brain systems (e.g. salience and central executive networks) in patients with ADHD (Cai, Chen, Szegletes, Supekar, & Menon, 2018; Shappell et al., 2021).

Existing brain dynamic studies in ADHD mainly rely on case–control study designs, testing for group-level differences between patients and controls, which are unable to capture heterogeneity within patients and their relation to clinical features (Satterthwaite et al., 2015). Recently, two neuroimaging meta-analyses of whole brain structural and functional studies of ADHD found no significant spatial convergence of altered gray matter volume and functional alterations in patients compared with healthy controls (Cortese, Aoki, Itahashi, Castellanos, & Eickhoff, 2021; Samea et al., 2019). They suggested the lack of significant spatial convergence may be accounted for heterogeneity of patients. Therefore, in recent years, there is growing support for a dimensional model of ADHD in which ADHD symptoms (e.g. inattentive and hyperactive/impulsive) are considered as continuously distributed from the general population to patients (Asherson & Trzaskowski, 2015; Singh & Rose, 2009). Moreover, there is a growing understanding that dimensional features of psychopathology have distinct relations to alterations in brain function that extend beyond patients whose conditions are sufficiently severe to meet diagnostic criteria (Clark, Cuthbert, Lewis-Fernandez, Narrow, & Reed, 2017; van Os, Linscott, Myin-Germeys, Delespaul, & Krabbendam, 2009). Notably, Shaw et al., found a pattern of cortical thinning occurred in typically developing children (TDC) who have varying levels of impulsive and hyperactive behaviors resembling that of children with ADHD (Shaw et al., 2011). Another study demonstrated reductions in cortical surface area in frontal, cingulate, and temporal regions in patients with ADHD that were linked to ADHD traits in the general population (Hoogman et al., 2019). Thus, there is potential merit in studying ADHD-linked features in both healthy individuals and those with ADHD.

A second issue is that discrete brain dynamic alterations can be associated with specific behavioral or cognitive features associated with the disorder. Cai et al., found that higher variability of cross-network interaction among default mode, salience, and central executive networks was correlated with inattention symptoms in ADHD (Cai et al., 2018), and Mowinckel et al., found increased DMN variability is related to cognitive impairments in ADHD (Mowinckel et al., 2017). While these early studies are promising, a full understanding of how local dynamic variability in brain function relates to different illness-associated behavioral and cognitive features requires further research (Insel et al., 2010).

Canonical correlation analysis (CCA) is a statistical tool for identifying relationships between two multi-dimensional variables, such as regional brain features and behavioral alterations in ADHD. This approach can evaluate the complex relationships between brain imaging data and clinical measures in a more integrated way than examining a large number of univariate correlations. In the present study, we applied an advanced machine learning technique called sparse CCA (sCCA) (Witten, Tibshirani, & Hastie, 2009) to simultaneously model multi-dimensional clinical and brain imaging measures and their relationships (Smith et al., 2015). Compared with traditional CCA, sCCA adds elastic net regularization to sparse features to be capable of discovering complex linear relationships between two high-dimensional datasets. sCCA has been successfully applied in studies of neurodegenerative diseases (Avants et al., 2014), psychopathology (Li et al., 2022; Xia et al., 2018), and brain-behavior relationships in healthy individuals (Smith et al., 2015).

To date, no study has explored the relationship among brain dynamics, cognitive functions, and behaviors to determine whether cognitive deficits mediate the relationship between dimensions of dynamic organization of brain function and behavioral features related to ADHD. Therefore, the present study aimed to delineate multivariate dFC patterns associated with ADHD-related behaviors and cognitive functions, and to clarify the relationship among them. First, we sought to establish relationships between distinct patterns of brain dynamics and behavioral and cognitive dimensions. Second, using mediation analysis, we tested the hypothesis that cognitive deficits mediate the relationship between brain dynamics and ADHD-associated behaviors.

## Materials and methods

### Participants and clinical assessments

A total of 161 right-handed children (6–16 years old) were recruited from local schools, including 85 drug-naive children with ADHD and 76 TDC. This study was approved by the local research ethics committee of West China Hospital of Sichuan University. All participants and their guardians provided written informed consent. Diagnosis of ADHD, and the absence of other Axis I psychiatric disorders, was determined by two experienced clinical psychiatrists using the Chinese Revision of the Patient Edition of the Structured Clinical Interview for DSM-IV (SCID-CR). TDC were screened using the SCID-CR Non-patient Edition to exclude individuals with any Axis I psychiatric diagnoses. This was done to better link findings specifically to ADHD. Also, clinical interviews were performed to exclude individuals with a known history of psychiatric illness in their first-degree relatives. Exclusion criteria for all participants included: (1) previous head trauma, neurologic disorders, or neurosurgery; (2) significant systemic physical illnesses; (3) maximum head displacement more than 1.5 mm or maximum rotation greater than 1.5° during resting-state functional magnetic resonance imaging (rfMRI) scanning; (4) inability to complete cognitive function tests; (5) full-scale IQ below 90 based on Wechsler Intelligence Scale for Children, Chinese Revision; and (6) contraindications to MR imaging. After data acquisition and quality control (online Supplementary Fig. S1), the final participant sample comprised 122 individuals (63 with ADHD; Table 1).

**Table 1.** Demographic and clinical characteristics of all participants in the present study

	Abbreviation	Mean ± s.d.	Range
Age (years)	/	10.3 ± 2.3	7–16
Number of boys	/	106	/
Number of girls	/	16	/
Corners' Parent Rating Scale			
Conduct problems	Con_C	9.1 ± 7.9	0–32
Study problems	Con_S	5.2 ± 3.6	0–12
Psychosomatic problems	Con_P	0.7 ± 1.2	0–5
Impulsivity-hyperactivity	Con_IH	4.1 ± 3.4	0–12
Anxiety	Con_A	1.3 ± 1.7	0–8
Hyperactivity index	Con_HI	9.4 ± 7.3	0–30
Achenbach Children Behavior Checklist			
Social withdrawn	CBCL_soc	3.3 ± 3.5	0–16
Somatic complaints	CBCL_S	1.3 ± 2.0	0–7
Anxious/Depressed	CBCL_AD	4.0 ± 4.3	0–19
Uncommunicative	CBCL_U	3.8 ± 3.3	0–12
Thought problems	CBCL_T	1.2 ± 1.8	0–7
Attention problems	CBCL_A	6.4 ± 4.5	0–18
Delinquent problems	CBCL_D	3.6 ± 3.3	0–17
Aggressive behaviors	CBCL_agg	10.0 ± 8.5	0–37
Internalizing	CBCL_I	8.5 ± 8.0	0–37
Externalizing	CBCL_E	13.7 ± 11.2	0–54
Visual Memory			
Immediate score	VM1	20.1 ± 3.3	7–24
Delayed score	VM2	19.2 ± 3.5	7–24
Stroop Test			
Stroop-C No. right	STRC_R	110.3 ± 1.9	103–112
Stroop-C No. error	STRC_E	1.4 ± 1.6	0–6
Stroop-C No. correction	STRC_C	1.0 ± 1.3	0–4
Stroop-C total score	STRC_S	111.2 ± 1.2	107–112
Stroop-C total time	STRC_T	82.4 ± 27.4	50–180
Stroop-CW No. right	STRCW_R	100.2 ± 9.6	72–112
Stroop-CW No. error	STRCW_E	10.7 ± 9.8	0–40
Stroop-CW No. correction	STRCW_C	5.5 ± 5.4	0–27
Stroop-CW total score	STRCW_S	104.0 ± 14.4	11–112
Stroop-CW total time	STRCW_T	221.9 ± 96.1	95–610
Fluency Test			
Word Total score	VFT_T	18.7 ± 5.9	8–42
Word No. error	VFT_E	0.1 ± 0.3	0–2
Word No. perseverative	VFT_P	0.7 ± 1.3	0–5
Word No. correct	VFT_C	17.7 ± 6.1	4–42
Ideational No. correct	IFT	13.4 ± 6.6	3–28
non-VFT No. correct	nVFT	15.8 ± 8.1	3–35
Wisconsin Card Sorting Test			
Total correct matches	WCST_C	33.3 ± 8.7	10–46
Total errors	WCST_E	11.7 ± 10.5	1–38
Perseverative errors	WCST_PE	3.0 ± 4.0	0–16
Noperseverative errors	WCST_NE	8.7 ± 7.2	1–29
Categories completed	WCST_cat	5.0 ± 1.9	0–7
Digital span (forward)	DS	8.1 ± 1.9	5–12

Abbreviations: s.d., standard deviation; Stroop-C/CW, Stroop Color/Color-Word Test; No., number of; VFT, Verbal Fluency Test.

Behavioral assessment included the Children Behavior Checklist (CBCL) and the revised Conners' Parent Rating Scale (CPRS), with ratings provided by parents. Cognitive testing included the Visual Memory Test of episodic memory (Temple, Davis, Silverman, & Tremont, 2006), the Stroop Test of inhibitory cognitive control (Jensen & Rohwer, 1966), the Wisconsin Card Sorting Test (WCST) of cognitive flexibility (Nelson, 1976), the Fluency Test used to evaluate the integrity of semantic systems (Shao, Janse, Visser, & Meyer, 2014), and forward Digital Span Test to assess short-term memory (Karatekin, 2004) (online Supplementary Methods 1.1 and Table 1). The behavioral and cognitive scores were continuously distributed from TDC to ADHD with considerable overlap (online Supplementary Fig. S2). Group differences of age, sex, and behavioral and cognitive scores are shown in online Supplementary Table S1, with children with ADHD generally showing more serious behavior problems and worse cognitive function than TDC. We then tested the main effect of diagnosis and sex, as well as the diagnosis × sex interactions on the cognitive or behavioral ratings (online Supplementary Table S2). We have also tested for group differences of cognitive or behavioral scores for boys and girls, as well as sex differences of cognitive and behavioral scores of TDC and ADHD, respectively (online Supplementary Table S3). For all participants, there was no sex difference in almost all cognitive and behavioral ratings, and there were no significant associations between age and behavioral ratings, but significant correlations between age and some scores of cognitive performance were found as expected (online Supplementary Table S4).

### MR data acquisition and preprocessing

All participants underwent rfMRI and high-resolution T1-weighted MR imaging by using a 3-T MRI system (Trio; Siemens). The rfMRI data were obtained with a gradient-echo echo-planar imaging sequence with the following parameters: repetition time (TR) = 2000 ms; echo time (TE) = 30 ms; flip angle = 90°; slice thickness = 5 mm with no gap, field of view = 240 × 240 mm^2^, matrix size = 64 × 64, voxel size = 3.75 × 3.75 × 5 mm^3^. Each brain volume comprised 30 axial slices to cover the whole brain, and each functional imaging session contained 205 volumes. High-resolution three-dimensional structural MRI was obtained with a magnetization prepared rapid gradient echo sequence (TR/TE = 1900/2.5 ms; flip angle = 9°; slice thickness = 1 mm; field of view = 256 × 256 mm^2^; matrix size = 256 × 256; number of sagittal slices = 176). During scanning, participants were instructed to keep their heads still and relax with their eyes closed without falling asleep or having systematic thought. Earplugs and foam padding were used to reduce noise and head motion.

MR image preprocessing was performed using the toolbox for Data Processing & Analysis of Brain Imaging (rfmri.org/DPABI). The first five volumes of rfMRI data were removed to reduce equilibration effects, leaving a total of 200 volumes for statistical analysis. The remaining functional images underwent slice-timing correction and were realigned to reduce displacement between volumes. Individual structural images were co-registered to the mean of functional images after realignment. The transformed structural images were then segmented into gray matter, white matter, and cerebrospinal fluid. To control for head motion and physiological noise, Friston-24 head motion parameters, linear trend, and signals from the white matter, cerebrospinal fluid, and global signal were regressed out. We performed global signal regression. Previous studies have suggested that the temporal variability of FC is sensitive to head motion induced artifacts (Laumann et al., 2017), and global signal regression is effective for reducing this problem (Lydon-Staley, Ciric, Satterthwaite, & Bassett, 2019). The Diffeomorphic Anatomical Registration Through Exponentiated Lie algebra tool was used to compute transformations from individual native space to the Montreal Neurological Institute template and to resample functional images to 3 × 3 × 3 mm^3^ resolution. Then, the normalized data were temporally filtered between 0.01 and 0.08 Hz. Participants with a maximum head displacement more than 1.5 mm or maximum rotation greater than 1.5° were excluded from the analysis.

### Dynamic functional network construction

**Fig. 1.**
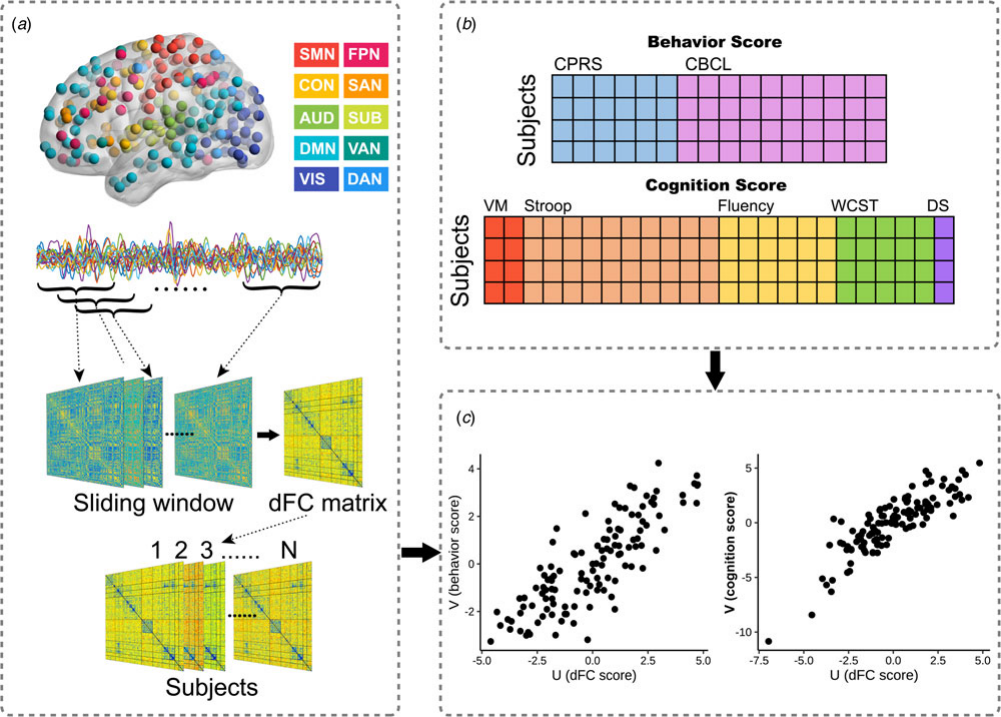
The schematic of sparse canonical correlation analysis (sCCA). (a): After preprocessing, time courses of resting-state functional magnetic resonance imaging data were extracted from 227 spherical regions of interest (ROIs) of the cortical and subcortical structures. ROIs of the same color belong to the same network as defined by Power et al. Using the sliding-window approach, we calculated the functional connectivity (FC) matrix of each time window. Then the dynamic FC (dFC) matrix of each subject was estimated by the standard deviation across all sliding windows. (b): Behavior and cognition scores were obtained, which were entered into sCCA as clinical features, respectively. (c): sCCA seeks a linear combination of dFC features and clinical features that maximize their correlation. Abbreviations: SMN, sensorimotor network; CON, cingulo-opercular network; AUD, auditory network; DMN, default mode network; VIS, visual network; FPN, fronto-parietal network; SAN, salience network; SUB, subcortical network; VAN, ventral attention network; DAN, dorsal attention network; CPRS, Corners' Parent Rating Scale; CBCL, Achenbach Children Behavior Checklist; VM, Visual Memory; WCST, Wisconsin Card Sorting Test; DS, Digital Span.

The dynamic functional network was built using the preprocessed rfMRI data. We partitioned the brain using the Power atlas (Power et al., 2011), excluding 33 brain regions that were not assigned to a specific network and four cerebellar regions. The remaining 227 brain regions are considered components of one of ten well-established large-scale resting-state networks (Mohr et al., 2016): the sensorimotor network (SMN), cingulo-opercular network (CON), auditory network (AUD), DMN, visual network (VIS), fronto-parietal network (FPN), salience network (SAN), subcortical network (SUB), ventral attention network (VAN), and dorsal attention network (DAN) (Fig. 1a). A sliding-window approach with 22-TR (44s) window size and one TR step was used to measure windowed FC matrices, as previous studies have suggested that time windows of 30s to 60s can successfully capture patterns of resting-state fluctuations of dFC (Luo et al., 2021; Preti, Bolton, & Van De Ville, 2017; Yin et al., 2022; You et al., 2022). The dFC matrices were estimated by the standard deviation across all windows between all pairs of brain regions (Fig. 1a). Higher dFC represents more temporal fluctuation and instability of FC across time windows.

To validate the consistency of dFC measures regardless of different methods, we used a flexible least squares (FLS) method in the DynamicBC toolbox (restfmri.net/forum/DynamicBC) (online Supplementary Methods 1.2). dFC matrices obtained using the FLS approach were very similar to those obtained by the sliding-window approach (online Supplementary Fig. S3).

### sCCA

We used sCCA to find relationships between high-dimensional brain imaging data and clinical data (Witten et al., 2009) (Fig. 1 and online Supplementary Methods 1.3). To limit the influence of potential confounders on sCCA results, we regressed age, sex, and mean framewise displacement out of dFC data, and regressed age and sex out of behavioral and cognitive data. Before sCCA, we used the Relief algorithm (Kira & Rendell, 1992) to select the top 1% of dFC features with the highest weight, and the selected dFC features were entered into dFC-behavior and dFC-cognition sCCA, respectively. We used elastic net regularization to determine the best sparsity parameters for computing the canonical correlations (online Supplementary Fig. S4). Permutation tests (1000 times) were used to assess the significance of the canonical correlations, and false discovery rate (FDR) correction was used to preserve experiment-wise Type I error rate at *p* < 0.05. We also explored the multivariate relationships between dFC and clinical assessments within each group, separately.

A bootstrapping procedure was used to estimate mean and standard error for canonical correlations and to identify reliable features that were consistently significant regarding the random resampling. We next calculated Pearson's correlation between each original variable and the corresponding canonical variate to confirm that these stable features were significantly related to their respective linear combined variates. Finally, to understand the meaning of each significant canonical variate and facilitate visualization, we calculated the sum of absolute loading values of dFC features at the network level (higher loadings indicating more importance of individual variates for corresponding canonical variates).

In order to validate whether different feature selection methods affect our sCCA result, principal component analysis was performed to reduce feature dimension. We also used dFC matrices obtained by the FLS methods to measure sCCA. These findings were similar to the main analysis (online Supplementary Figs S6 and S7).

### Mediation analysis

Pearson correlations were computed to identify significant relationships between pairs of dimension scores (linear combination scores) for cognition and behavior. The correlation analysis revealed that the inhibition and flexibility cognitive dimension score significantly correlated with inattention/hyperactivity dimension score (*r* = −0.429, *p* < 0.001), while the other three pairs didn't show a significant correlation. Therefore, we inferred that cognitive functions involving inhibitory control and cognitive flexibility could mediate the relationship between dFC and inattention/hyperactivity. We selected overlapping dFCs significantly correlated to both the cognition and behavior dimensions for the mediation analysis. Then we calculated the mean value of all dFCs positively or negatively correlated with the cognition dimension score respectively to represent two types of overlapping dFCs (named positive links and negative links that mean dFCs with positive/negative loadings in canonical correlation, respectively). We treated overlapping dFCs as independent variables, cognitive function as a mediator, and behavioral problems as the outcome. Covariates used in this model included age and sex. The total effect, direct effect, and indirect effect were estimated (online Supplementary Methods 1.4).

## Results

### Canonical correlation patterns

#### Linked dimensions of behavior/cognition and dFC

**Fig. 2.**
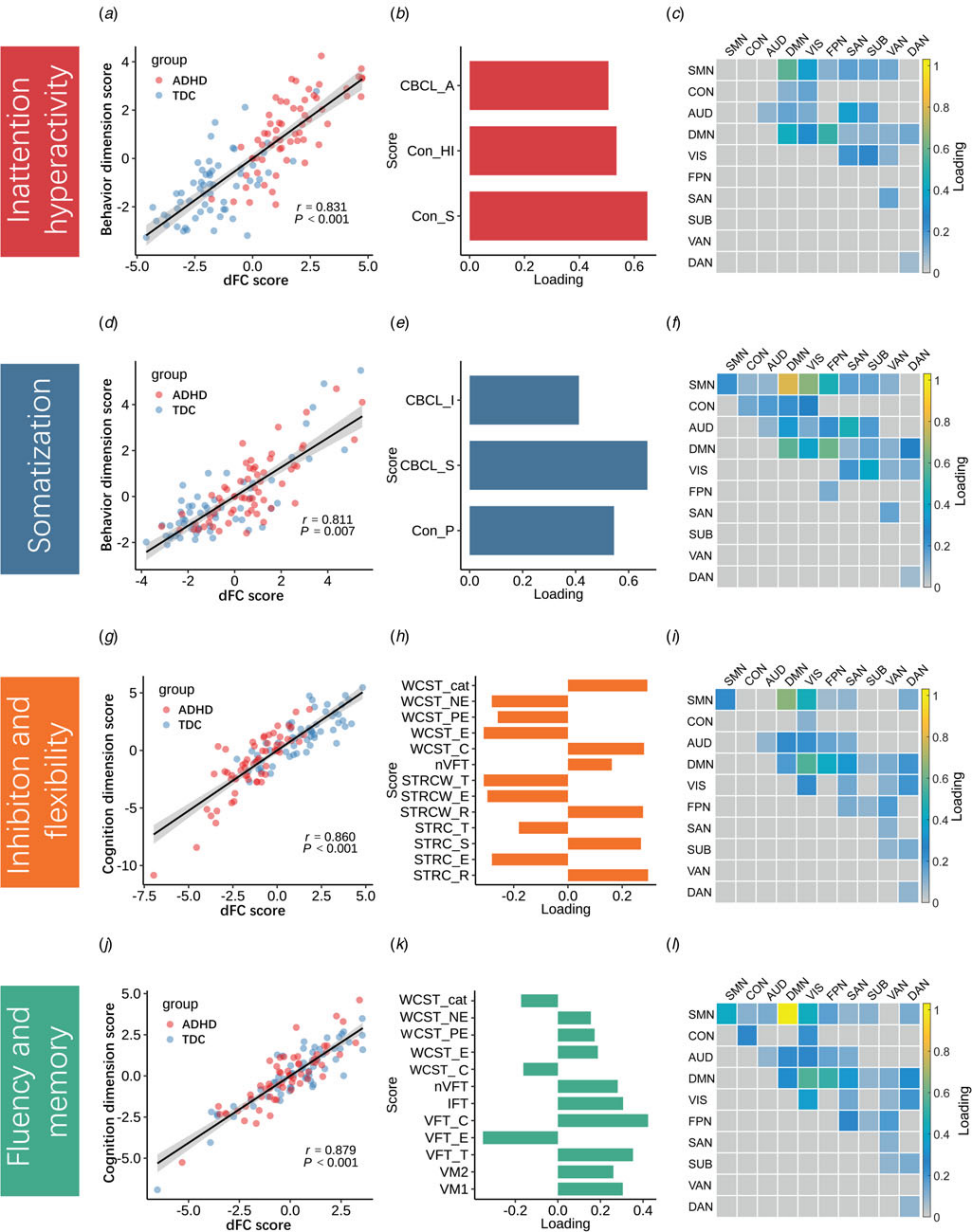
Scatter plot and contributions of behavior/cognition and dynamic functional connectivity (dFC) within/between networks for each significantly linked dimension. (a, d, g, j): The scatter plots of linear combinations of dFC and behavior/cognition scores, respectively, demonstrate the correlated multivariate patterns of dFC and clinical variates. (b, e, h, k): The loadings of clinical variates for each dimension. (c, f, i, l): The sum of the absolute values of loadings of dFC at the within- and between-networks level. For the abbreviations of clinical measurements and brain networks see Table 1 and Fig. 1.

Each canonical variate was determined by a weighted set of dFC features with cognitive or behavioral features (Fig. 2 and online Supplementary Results 2.2). For dFC-behavior relationships as examined using sCCA, two canonical variate pairs were significantly correlated. Based on the highly weighted item compositions, the two significant behavioral dimensions (canonical variates) represented behavioral traits of ‘inattention/hyperactivity’ and ‘somatization’ (*r*_mean_ = 0.857, *p*_FDR_ < 0.001; *r*_mean_ = 0.836, *p*_FDR_ = 0.042, respectively) (online Supplementary Fig. S5b). For dFC-cognition sCCA, two pairs were significant, the two significant cognitive dimensions represented cognitive traits of ‘inhibition and flexibility’ and ‘fluency and memory’ based on their highly weighted item compositions (*r*_mean_ = 0.864, *p*_FDR_ < 0.001; *r*_mean_ = 0.855, *p*_FDR_ < 0.001, respectively) (online Supplementary Fig. S5d). Then, we applied the loading values obtained from all participants to ADHD and TDC separately and found there was no significant difference in the canonical correlation coefficient of the significant canonical variate pairs between ADHD and TDC (online Supplementary Table S6). We performed sCCA in TDC and ADHD groups separately and found quite similar dFC patterns in the two groups for each behavioral and cognitive dimension, and the dFC patterns in each group resembled those obtained when all participants were considered together (online Supplementary Table S7).

#### Characteristics of linked dimensions

**Fig. 3.**
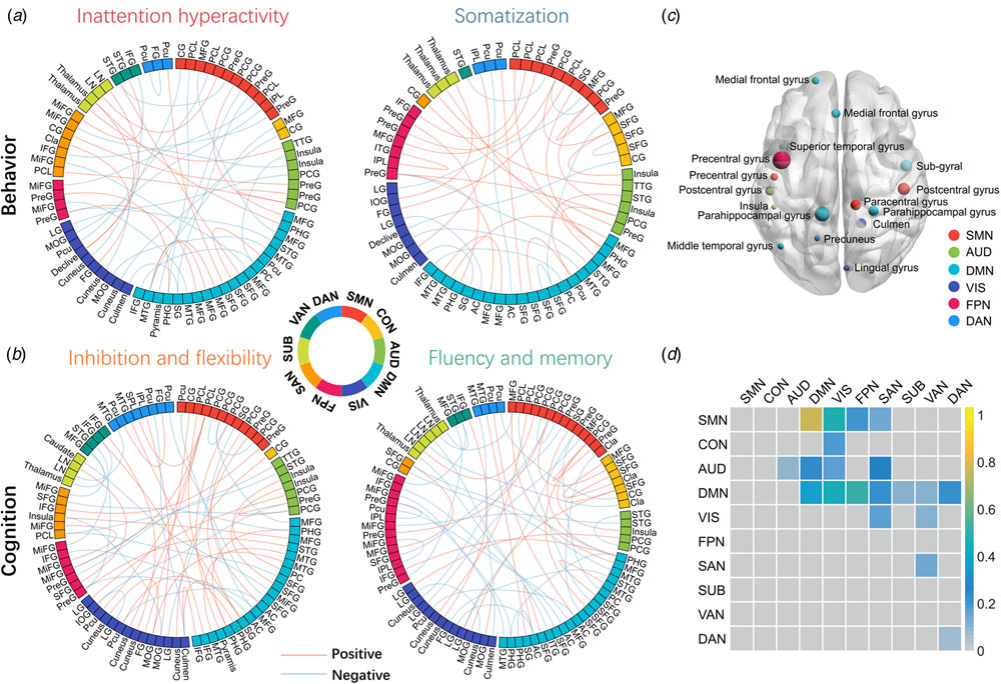
Both positive and negative loadings for specific dynamic functional connectivity (dFC) links contributed to each behavior dimension (a) and to each cognition dimension (b). By searching for the overlapped dFC that contributed significantly to different dimensions, we found overlapped nodes (c) and dFC shown at the network level (d) that significantly contributed to every dimension. The size of the spheres in panel C denotes the sum of their absolute loadings. In panel D, the colored grids represent the sum of the absolute loadings of shared dFCs at the network level, and more yellow color of the grid represents the higher mean value of network-level contribution for all dimensions. For the abbreviations of brain networks see Fig. 1 and for the abbreviations of specific brain areas in panels A and B see online Supplementary Table S5.

The inattention/hyperactivity dimension was comprised of factor scores including hyperactivity index, attention problems, and study problems (loading value: *v* = 0.535; 0.506; 0.648, respectively) (Fig. 2b). Scores on each of these three factors were stable and strongly correlated with the canonical variate representing inattention/hyperactivity (online Supplementary Fig. S9a). At the network level, the inattention/hyperactivity behavior dimension mainly correlated with dFC within DMN (sum of absolute loading value: sum_u = 0.425) and between DMN and other networks, especially in the following pairs: DMN-SMN (sum_u = 0.567) and DMN-FPN (sum_u = 0.509) (Fig. 2c). Positive loadings of dFC within DMN and negative loadings of dFC between DMN and SMN were most prominent (Fig. 3a and online Supplementary Fig. S10a).

The somatization dimension was composed of psychosomatic problems, somatic complaints, as well as internalizing features (*v* = 0.545; 0.671; 0.412, respectively). Their loading value and correlations with the canonical variate are shown in Fig. 2e and online Supplementary Fig. S9b. This behavior dimension was mainly associated with dFC within DMN (sum_u = 0.572) and SMN (sum_u = 0.215), as well as between the two networks (sum_u = 0.791) and between SMN and VIS (sum_u = 0.648) (Fig. 2f). This dimension was characterized by positive loadings of dFC within DMN and SMN, as well as both positive and negative loadings of dFC between DMN and SMN regions (Fig. 3a and online Supplementary Fig. S10b).

Analysis of cognitive functions identified an inhibition and flexibility dimension that was comprised of Stroop and WCST scores. Stroop-CW total time (*v* = −0.310) and WCST number of errors (*v* = −0.311) contributed most to this dimension (Fig. 2h and online Supplementary Fig. S9c). The fluency and memory dimension captured the association between Verbal Fluency Test (VFT) scores and Visual Memory (VM) test scores (Fig. 2k and online Supplementary Fig. S9d). Both cognitive dimensions correlated with dFCs within SMN (sum_u = 0.240; 0.413), and within the VIS (sum_u = 0.231; 0.359) and DMN networks (sum_u = 0.178; 0.293). Associations of these three networks with each other and with other networks were also identified, chiefly including dFCs between SMN-DMN (sum_u = 0.655; 1.031), SMN-VIS (sum_u = 0.447; 0.413), DMN-VIS (sum of u = 0.556; 0.556), and DMN-FPN (sum_u = 0.404; 0.513) (Fig. 2i and l). At the single dFC link level, the two dimensions were characterized by positive loadings of dFC within DMN and between DMN and SMN/VIS as well as negative loadings of dFC between DMN and FPN (Fig. 3b, online Supplementary Fig. S10c, and d).

There were several features shared across all dimensions. At the network level, shared dFCs were mainly located within DMN and between DMN and other networks (Fig. 3d). These dFCs could be mapped to specific nodes, and 16 brain regions consistently contributed to all four dimensions, in which seven belonged to DMN, and three belonged to each of SMN and VIS (Fig. 3c).

### Mediation analysis

**Fig. 4.**
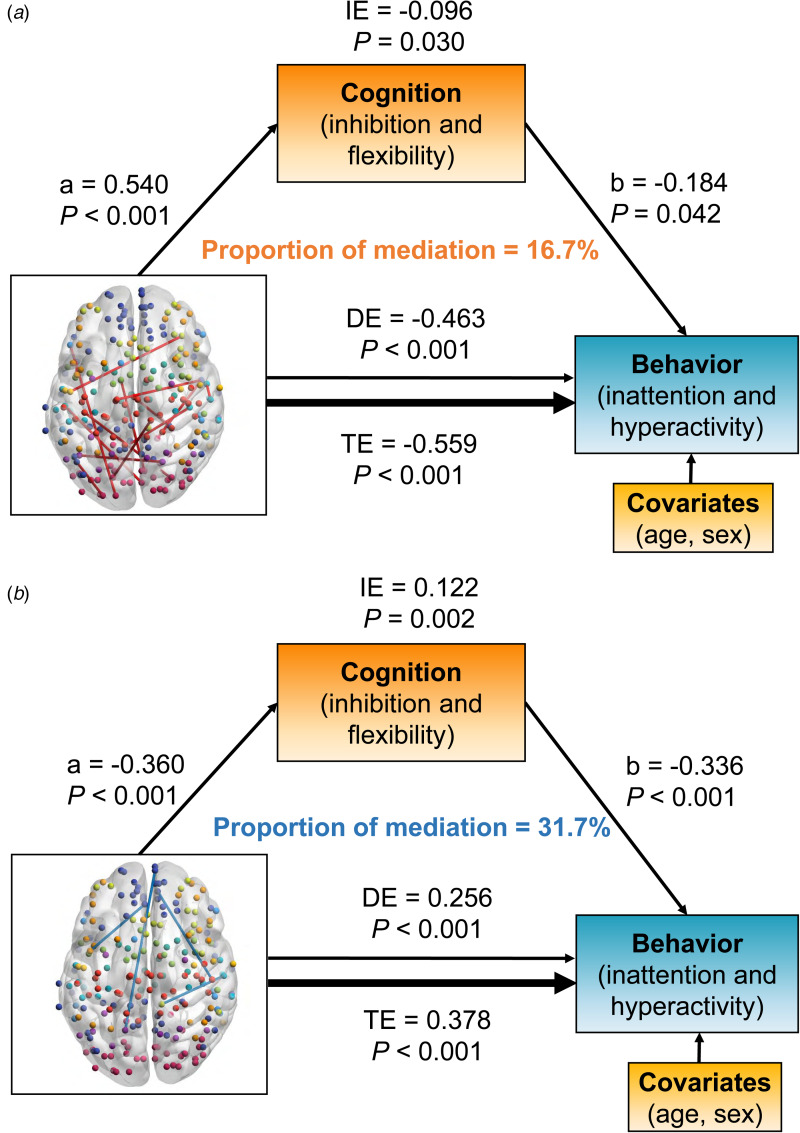
The significant results of mediation analysis showed that the overlapped dynamic functional connectivity (dFC) with negative (a) and positive (b) links on behaviors of inattention and hyperactivity through cognition of inhibition and flexibility, respectively. Path a is the direct effect of dFC on cognition, and path b is the direct effect of cognition on behaviors. DE (direct effect) is the direct effect of dFC on behaviors after controlling for cognition. IE (indirect effect) is the mediation effect of dFC on behaviors through cognition. TE (total effect) is the total effect of DE and IE. Covariates used in the models included age and sex.

The relationships between dFC and behavioral ratings of inattention/hyperactivity were mediated by cognitive functions of inhibition and flexibility (Fig. 4). Positive links were mainly located between DMN and SMN or VIS (Fig. 4a), while negative links were mainly located between DMN and FPN (Fig. 4b).

Positive links had an inverse association with behavior problem severity [total effect = −0.559, 95% CI (−0.706 to −0.400), *p* < 0.001]. The inhibition/flexibility dimension had significant indirect effects (IE) on the relationship between positive dFC links and behavioral features of inattention/hyperactivity [IE = −0.096, 95% CI (−0.188 to −0.010), *p* = 0.030], and the proportion of mediation explained for this relationship was 16.7% (Fig. 4a). Interestingly, the relationship between negative links and the behavioral dimension were also mediated by inhibition and flexibility [total effect = 0.378, 95% CI (0.212–0.540), *p* < 0.001]. The mediation effect of inhibition and flexibility on these dFC-behavior relations was significant [IE = 0.122, 95% CI (0.048–0.220), *p* = 0.002], and the proportion of mediation explained was 31.7% (Fig. 4b).

## Discussion

In the present study, we examined the clinical relevance of dFC for cognitive and behavioral features of ADHD. We identified four specific patterns of dynamic brain activity within and between well-established brain networks, each related to a different cognitive or behavioral dimension of ADHD. These effects were most consistently seen in the DMN. Our work provides potential markers for the identification of children with ADHD likely to demonstrate specific behavioral or cognitive problems, but more importantly helps explain how specific functional brain features contribute to specific behavioral features of the disorder. It is important to note that similar patterns of brain-behavior/cognition relations, though not the severity of the alterations, were seen in children with ADHD and healthy controls. This finding highlights the dimensional presentation of these associations in youth regardless of whether individuals meet the criteria for ADHD. Further, we observed that the cognitive functions of inhibition and flexibility mediated the association between brain functional dynamics and behaviors of inattention and hyperactivity. This is consistent with the model that functional brain alterations in ADHD disrupt neurocognitive processes in ways that contribute to the behavioral characteristics that define the disorder. Overall, our findings demonstrate that distinct patterns of alterations in the dynamic activity within and between brain networks contribute to different behavioral and cognitive features of ADHD.

### dFC patterns correlate with specific behavior and cognitive dimensions

In the sCCA examining relations between dFC and behavioral data, two canonical variates pairs were significant. The first pair reflected a specific pattern of dFC features related to attention problems and hyperactivity. The dFC pattern that was most strongly associated with this dimension included positive loadings of dFC within DMN and negative loadings of dFC between DMN and SMN or FPN. In accordance with our results, Lin et al., adopted a similar computational approach but their analysis was based on static FC data. They found that FC patterns most strongly related to the inattention hyperactivity behavioral dimension mainly included hyperconnections between DMN and other networks (Lin et al., 2018).

Mounting evidence indicates that alterations in DMN function are associated with ADHD (Kaboodvand, Iravani, & Fransson, 2020), particularly in relation to attention lapses (Castellanos & Aoki, 2016; Weissman, Roberts, Visscher, & Woldorff, 2006). A previous study found that increased temporal variability of FC within DMN was related to reduced cognitive performance in patients with ADHD (Mowinckel et al., 2017). Increased temporal fluctuation of FC within DMN indicates that the FC among nodes of DMN is more variable over time. The functional instability of DMN nodes may reduce its temporal coherence and functional interactions with other networks, as we observed less variable dynamic cross-network interaction between DMN and SMN or FPN. Importantly, these two task-active networks mediate motor reactivity to sensory input and attention to external events, both of which are key areas of disturbance associated with ADHD. This interpretation is consistent with our findings, as we observed that decreased dFC between DMN and SMN/FPN was related to more severe inattention and hyperactivity.

Another significant canonical variates pair captured the association between somatization and a brain dynamic pattern that mainly involved dFC within and between SMN and DMN, as well as between those two networks and other networks (e.g. SMN-VIS, DMN-FPN). The dimension of somatization was composed of psychosomatic problems, somatic complaints, and internalizing. Some prior static FC analyses reported findings consistent with our findings related to dynamic changes in FC. For example, Ernst et al., found that lower static connectivity of DMN predicted higher internalizing symptoms in a healthy population (Ernst et al., 2019). Previous studies have shown that connections between SMN and DMN are related to somatization (Otti et al., 2013; Zhao et al., 2017). One possible explanation for this relationship is that abnormal dynamic interactions between SMN and DMN may induce an experience of endogenous bodily experiences without peripheral pathology.

For the analysis of canonical correlates between dFCs and cognitive functions, two cognition dimensions were correlated with dFC patterns mainly characterized by dFC within SMN, VIS, and DMN, as well as among the three networks. Difficultly ignoring extraneous stimuli is one of the core clinical deficits of ADHD, and the DMN plays an important role in regulating the allocation of attention between external stimuli and internal mental activities (Silberstein, Pipingas, Farrow, Levy, & Stough, 2016). Flexible shifts of brain function between the DMN and other networks are known to support sensorimotor and higher cognitive functions including cognitive flexibility and working memory (Chadick & Gazzaley, 2011; Dajani & Uddin, 2015; Fornito, Harrison, Zalesky, & Simons, 2012; Sweeney et al., 1996; Takeuchi et al., 2015; Vatansever, Menon, Manktelow, Sahakian, & Stamatakis, 2015). Previous fMRI studies have demonstrated that patients with ADHD showed deactivation of occipital regions during spatial tasks (Vance et al., 2007) and occipital hyperactivations during the performance of behavioral inhibition, working memory, and attentional tasks (Dillo et al., 2010; Hale, Bookheimer, McGough, Phillips, & McCracken, 2007). Interestingly, we found dynamics of FC between DMN and SMN or VIS positively correlated with cognitive function, indicating that more flexible functional communication among the three brain networks was related to better cognitive function.

Altered activity within the DMN was a common feature across the four canonical pairs identified. In support of this finding, a comprehensive meta-analysis of task-based fMRI studies found that children with ADHD showed hyperactivation during tasks within DMN (Cortese et al., 2012). As previously suggested, disrupted connectivity within the DMN may alter interactions of DMN with task-based networks, and perhaps also the ability to reduce DMN function when task-active networks are required for current cognitive demands. Such alterations could contribute to the disruptions in cognition and behavior seen in ADHD (Kaboodvand et al., 2020; Rolls et al., 2021). Specially, we observed higher dFCs between DMN and low-order networks (brain areas responsible for sensory and sensorimotor processes, e.g. VIS, SMN) (Liu et al., 2012) mainly correlated with fewer behavior problems and better cognitive performance as discussed above. Our results showed that higher dFCs between DMN and high-order networks (e.g. FPN) mainly correlated with more serious behavioral problems and poorer cognition, which is consistent with a previous study (Cai et al., 2018). While higher dFCs between DMN and high-order networks may lead to a reduced ability to effectively recruit regional activity to address behavioral demands, and thus contribute to the disruptions of cognitive function and the emergence of behavioral problems related to ADHD. Previous meta-analyses of diffusion tensor imaging studies of ADHD revealed lower fractional anisotropy values in ADHD compared with TDC in the corpus callosum, inferior fronto-occipital fasciculus, and inferior longitudinal fasciculus (Aoki et al., 2018), which might represent structural underpinning of altered dFCs. This possibility warrants investigation in future research.

### The relationships among dFC, cognitive function, and behavior

Mediation analysis indicated that the association between dFC and inattention/hyperactivity ratings was mediated by cognitive abilities of inhibitory control and cognitive flexibility. Inhibitory control and cognitive flexibility have been suggested to be areas of basic neuropsychological deficits in ADHD (Castellanos & Tannock, 2002; Smith, Taylor, Brammer, Toone, & Rubia, 2006). This concept together with our results implies that etiological factors of ADHD affect inhibitory control and cognitive flexibility, and are important factors leading to the manifestation of the ADHD phenotype.

More specifically, increased dynamic interaction between DMN and high-order networks was associated with more serious behavior problems. These relationships were in part mediated by disruptions in the cognitive functions of inhibition and flexibility. These findings demonstrate a complex pattern of dFC associated with cognitive functions, the disruption of which contributes to the behavior features of ADHD. Moreover, our finding that cognitive function mediated brain-behavior relationship may provide a rationale for the use of cognitive therapies focusing on cognitive flexibility and the cognitive control of automatic response tendencies for the treatment of ADHD, as reducing those deficits may lead to improved behavioral outcomes (Young, Moghaddam, & Tickle, 2020).

### Limitations

Several potential limitations to our study should be considered. First, we didn't replicate our findings in an independent sample. However, we note that our validation analysis using a different method to quantify dynamics of FC indicated that dFC alterations were not a result of the specific method and window size choice used in our primary analyses. Moreover, in the sCCA analysis, we used different feature selection methods yet obtained similar results. Second, although we adopted one of the most popular brain parcellation schemes used in previous studies, using a different brain atlas may influence detected dFC patterns. However, most of our findings of relations between regional brain function and behavior relevant to ADHD are consistent with the previous literatures. Third, further work is needed to examine developmental and sex-specific patterns of brain-behavior/cognition patterns associated with ADHD, and the degree that they vary over time in relation to the severity of symptoms and treatment initiation. Fourth, our current analysis only considered dFC. Future research might benefit from incorporating multi-modal imaging features and genomics to more fully understand the mechanisms responsible for the brain-behavior/cognition patterns identified in the present study (Luo, You, DelBello, Gong, & Li, 2022; Zhao et al., 2022). Fifth, in clinical samples, comorbidity in patients with ADHD is common, and patients are typically receiving medication treatments for ADHD. In our sample, the children with ADHD were all drug-naive and free from any psychiatric comorbidities. While this helps link our findings directly and specifically to ADHD features, it may also limit the generalization of the present results to the general population of patients with ADHD. Finally, the IQ cut-off in our study is 90 to avoid non-ADHD related sources of cognitive impairment, which also may limit the generalizability of our findings.

## Conclusion

In summary, we identified four multivariate patterns of brain dFC that were each highly correlated with distinct dimensions of behavior and cognition. The dFC within DMN and in relation of DMN with other networks was common to all identified brain-behavior/cognition dimensions, highlighting the importance of DMN alterations in ADHD. Mediation analysis revealed that cognitive functions of inhibition and flexibility mediate the relationship between brain dynamics and behavioral features of inattention and hyperactivity. These results provide novel insights into the complexity of brain function alterations in ADHD, and the distinct relation of specific patterns of altered regional dFC with different cognitive and behavioral aspects of ADHD.

## Data Availability

The data that support the findings of this study are available from the corresponding authors upon reasonable request.

## Supplementary material

For supplementary material accompanying this paper visit https://doi.org/10.1017/S0033291723000089. click here to view supplementary material
