# Supplementary material for: Patterns of brain dynamic functional connectivity are linked with attention-deficit/hyperactivity disorder-related behavioral and cognitive dimensions
Source: Psychol Med. 2023 Feb 7;53(14):6666–77. doi: 10.1017/S0033291723000089 (PMC10600939; doi:10.1017/S0033291723000089)
Supplement: Supplementary file 1 [file S0033291723000089sup001.docx]

**Supplementary Materials**

**Patterns of brain dynamic functional connectivity are linked with attention-deficit/hyperactivity disorder-related behavioral and cognitive dimensions**

[**1. Supplementary Methods** 3](#_Toc119873918)

[**1.1 Clinical assessment** 3](#_Toc119873919)

[*Children Behavior Checklist (CBCL)* 3](#_Toc119873920)

[*Conners’ Parent Rating Scale (CPRS)* 3](#_Toc119873921)

[*Visual Memory Test* 3](#_Toc119873922)

[*Stroop Test* 4](#_Toc119873923)

[*Fluency Test* 4](#_Toc119873924)

[*Wisconsin Card Sorting Test (WCST)* 5](#_Toc119873925)

[*Digital Span Test (forward)* 5](#_Toc119873926)

[**1.2 Validation of brain dynamic functional networks** 5](#_Toc119873927)

[**1.3 Sparse canonical correlation analysis (sCCA)** 6](#_Toc119873928)

[*Feature dimension reduction* 7](#_Toc119873929)

[*Calculate sparse canonical correlation* 8](#_Toc119873930)

[*Permutation test* 9](#_Toc119873931)

[*Resampling procedure* 9](#_Toc119873932)

[*Interpretation of canonical variates* 10](#_Toc119873933)

[**1.4 Mediation analysis** 10](#_Toc119873934)

[**2. Supplementary Results** 11](#_Toc119873935)

[**2.1 The diagnosis*sex interactions on behavioral and cognitive scores.** 11](#_Toc119873936)

[**2.2 Canonical correlation patterns between dFC and clinical assessments** 12](#_Toc119873937)

[*Linked dimensions of canonical variates* 12](#_Toc119873938)

[**3. Supplementary Tables** 14](#_Toc119873939)

[**Table S1.** Demographic and clinical characteristics of children with attention-deficit/hyperactivity disorder (ADHD) and typically developing children (TDC). 14](#_Toc119873940)

[**Table S2.** The main effects of diagnosis and sex, as well as the diagnosis*sex interactions on the cognitive or behavioral ratings. 16](#_Toc119873941)

[**Table S3.** Diagnostic group differences of cognitive or behavioral scores for boys and girls, as well as sex differences of cognitive or behavioral scores of typically developing children (TDC) and children with attention-deficit/hyperactivity disorder (ADHD). 18](#_Toc119873942)

[**Table S4.** Differences in clinical scores between different sexes and correlations between age and clinical scores in all participants. 21](#_Toc119873943)

[**Table S5.** The information about the 227 brain regions of the Power atlas used in our study. 23](#_Toc119873944)

[**Table S6.** The multivariate correlation coefficients (*r*) within TDC and ADHD group for each significant dimension, separately, as well as the statistical between-group differences of *r*. 29](#_Toc119873945)

[**Table S7.** The dynamic functional connectivity patterns for each behavioral and cognitive dimension within TDC and ADHD, respectively. 30](#_Toc119873946)

[**4. Supplementary Figures** 32](#_Toc119873947)

[**Fig. S1.** The pipeline of participants recruitment. Abbreviation: ADHD, Attention-Deficit/Hyperactivity Disorder. 32](#_Toc119873948)

[**Fig. S2.** The scatter plots and boxplots show the distribution of behavioral (A) and cognitive (B) scores for typically developing children (TDC) and children with Attention-Deficit/Hyperactivity Disorder (ADHD). See Table 1 for abbreviations of behavioral and cognitive measurements. 33](#_Toc119873949)

[**Fig. S3.** The dynamic functional connectivity (dFC) matrices obtained using a sliding-window approach were very similar to those defined using the flexible least squares (FLS) approach. 34](#_Toc119873950)

[**Fig. S4.** Sparsity parameter search for sparse canonical correlation analysis (sCCA). 35](#_Toc119873951)

[**Fig. S5.** Sparse canonical correlation analysis (sCCA) captured multivariate patterns of linked dimensions of dynamic functional connectivity (dFC) patterns and behavior or cognition (which were the main results in the present study). 36](#_Toc119873952)

[**Fig. S6.** Validation analysis: sparse canonical correlation analysis (sCCA) results by using principal component analysis for feature dimension reduction, which was calculated using the sliding-window approach. 37](#_Toc119873953)

[**Fig. S7.** Validation analysis: sparse canonical correlation analysis (sCCA) results by using flexible least squares (FLS) method to measure dynamic functional connectivity (dFC) and Relief algorithm for feature dimension reduction. 38](#_Toc119873954)

[**Fig. S8.** A bootstrapping approach was used to identify stable clinical features contributing to each linked dimension. 39](#_Toc119873955)

[**Fig. S9.** The correlation between each original clinical variable and the corresponding canonical variate in the main results. 40](#_Toc119873956)

[**Fig. S10.** The matrices of pie charts denoting contribution proportion of within- or between-networks dynamic functional connectivity (dFC) with positive and negative loadings, respectively, for each dimension. 41](#_Toc119873957)

[**References** 42](#_Toc119873958)

**1. Supplementary Methods**

**1.1 Clinical assessment**

*Children Behavior Checklist (CBCL)*

CBCL is a well-established questionnaire on which parents rate a child’s behavior as 0 (not true), 1 (somewhat or sometimes true), or 2 (very often true or often true) for each item. These behaviors are classified into the following factors: social withdrawal, somatic complaints, anxious/depressed, uncommunicative, thought problems, attention problems, delinquent problems, aggressive behaviors, internalizing, and externalizing.

*Conners’ Parent Rating Scale (CPRS)*

The CPRS is a questionnaire used to evaluate problematic behaviors such as temper, sleep, and peer relationships. The parent rates each behavior on a four-point Likert-type scale. For CPRS, behaviors are aggregated into the following factors: conduct problems, study problems, psychosomatic problems, impulsive-hyperactive behaviors, anxiety, and hyperactivity index.

*Visual Memory Test*

Visual Memory Test is part of the Wechsler Memory Scale, including both immediate and delayed performance. Two designs were presented individually for 10 seconds. Following each presentation, the participants were asked to draw the designs from memory. After completion of the designs, the participants were instructed to remember the designs for later recall. After a delay of 30 minutes, the participants were asked to reproduce the designs from memory again. The scores on immediate recall and delayed recall were recorded.

*Stroop Test*

The Stroop Test consists of two conditions completed in a fixed order: naming colors (Color Test, Stroop-C), and incongruent color naming of color words (Color-Word Test, Stroop-CW). In the color condition, participants were asked to name the colors (red, blue, green, or yellow) of the presented words with congruent colors. The color-word condition consists of colored words in incongruent colors. In the color-word condition, participants were asked to name the color of the ink instead of reading the word. The dependent variables of the Stroop Test include number right, number error, number correction, and total time. The Stroop Test is used to measure inhibitory control.

*Fluency Test*

Fluency is evaluated as the ability to search an internal semantic network to produce words that start with a letter or are in a class (such as animals). Cognitive processes involved in verbal fluency performance include processing speed, depth of vocabulary, semantic memory, inhibition, and set maintenance (Sergeant, Geurts, & Oosterlaan, 2002). Participants are provided one minute to name as many words as they can for the given category.

The ideational fluency task was based on the Used Objects Test. Participants were asked to generate possible uses of three objects for one minute. The total number of generated correct responses was recorded. The responses from the three objects were combined to obtain a summary score. The non-verbal fluency task, also called Design Fluency, requires that subjects come up with as many objects that have the same shape as the given geometric form. This task was scored by counting the total number of novel responses.

*Wisconsin Card Sorting Test (WCST)*

In the WCST, participants are asked to change sorting strategies after correctly matching a card according to a certain stimulus feature (color, number, or form) for ten consecutive trials. Performance measures include perseverative errors, non-perseverative errors, total errors, and categories completed. The WCST mainly reflects cognitive flexibility and set-shifting.

*Digital Span Test (forward)*

In the forward Digital Span Test, participants are presented a series of digits and are then asked to recall the sequence of numbers correctly, with increasingly longer digit sequences being tested in each trial. The longest number of digits that could be recalled correctly was recorded as the measure of the participant’s memory span. This test is used to measure short-term memory.

**1.2 Validation of brain dynamic functional networks**

In order to validate the consistency of dynamic functional connectivity (dFC) measures regardless of window size, we used a flexible least squares (FLS) method in DynamicBC toolbox (restfmri.net/forum/DynamicBC) to evaluate the brain dynamics. Which can be used to estimate the continuously changed model parameters at each observation (e.g. time point). To describe the dynamic connection among the brain regions, a time-varying parameter regression method was used, the formula is as follows:

*y*(*t*) = *x*(*t*)*β*(*t*) + *u*(*t*)

in which *x*(*t*) and *y*(*t*) are the signals of seed and target brain regions at time point *t*, and *β*(*t*) is the regression coefficient that reflects the relationship between *x* and *y* at time *t*, and *u*(*t*) is the approximation error. The basic idea of FLS is to minimize the total sum of two types of residual errors. The first one is the sum of squared residual errors, in which *y*(*t*) - *x*(*t*)*β*(*t*) $\approx0$:

$r_{M}^{2}$(*β*, *T*) = $\sum_{t=1}^{T} (y\left( t \right)-x(t)\beta(t))$^2^

The other one is the sum of squared residual dynamic error, in which FLS declares that the vector of coefficients evolves slowly over time$(\beta\left( t+1 \right)-\beta\left( t \right)\approx0)$, the formula is shown as follows:

$r_{D}^{2}$(*β*, *T*) = $\sum_{t=1}^{T} (\beta\left( t+1 \right)-\beta\left( t \right))$^2^

With a given *μ* weighting parameter, Kalaba et al. (Kalaba & Tesfatsion, 1989) defined the incompatibility cost assigned to any $\beta$ coefficient sequence as follows:

*C*(*β, μ, T)* = *μ*·$r_{D}^{2}$(*β*, *T*) + $r_{M}^{2}$(*β*, *T*)

The incompatibility cost function *C*(*β, μ, T)* generalizes the goodness-of-fit criterion function for ordinary least squares estimation by permitting the coefficient vector *β*(*t*) to vary over time. When *μ* was set to zero, $r_{M}^{2}$ can generally be bought down close to zero and the corresponding value for $r_{D}^{2}$ will be relatively large, resulting in a rather erratic sequence of estimates. As *μ* becomes relatively large, the incompatibility cost function assigns all importance to the dynamic specification. This case yields the ordinary least squares solution, $r_{M}^{2}$ is minimized subject to the following formula: $r_{D}^{2}$ = 0. After obtaining the coefficient vector *β* for each time point, the temporal variability was then be estimated by the standard deviation across all time points of the whole scanning between each pair of brain regions. The dFC matrices obtained by FLS approach were very similar to that of sliding-window approach in the present study (Fig. S3).

**1.3 Sparse canonical correlation analysis (sCCA)**

Using unsupervised sCCA allows continuous dimensions of psychopathology to be present in a varying degree, and related to distinct patterns of brain function. In these analyses, dimensional features of psychopathology of an individual are represented as patterns of regional brain dynamic abnormalities, which together produce specific combinations of abnormal behaviors and cognitive functions.

*Feature dimension reduction*

Each dFC matrix comprised very large feature dimensions (227 × 226/2 = 25651). To reduce the feature dimensionality of the dFC matrices, we used Relief algorithm (Kira & Rendell, 1992) to select the top 1% of features with the highest weights. As an individual evaluation filtering feature selection method, Relief calculates a proxy statistic for each feature that can be used to estimate feature “importance” for the target concept. These feature statistics are referred to as feature weights or “scores” that range in the interval [-1, 1], with higher “scores” indicating having more “importance”. The importance of a feature in the Relief algorithm is based on the ability of using the feature to distinguish short-distance samples. Relief identifies two nearest neighbor instances of the sample *R*; one with the same class as the target, called the *nearest hit* (*H*) and the other with the opposite class, called the *nearest miss* (*M*). Then update the weight of each feature according to the following rules: if the distance between *R* and *H* on a feature is less than the distance between *R* and *M*, it means that the feature is useful for distinguishing the nearest neighbors of the same type from different types, then increase the weight of the feature; conversely, if the distance between *R* and *H* on a feature is more than the distance between *R* and *M*, indicating that the feature has a negative effect on distinguishing the nearest neighbors of the same type from different types, then reduce the weight of the feature. For each feature, the above process was repeated and finally the weights of all features were obtained.

To validate whether different feature selection methods will affect our sCCA result, an alternative approach for feature dimension reduction is principal component analysis (PCA). From PCA we selected the top 111 components (explaining 95% of the variance for dFC features) as features entered into dFC-behavior and dFC-cognition sCCA, respectively. We mainly showed the results of Relief because it allowed direct use of primary dFC features, instead of latent variables (principal components from PCA), as the input features to sCCA, thus increasing the interpretability of the results. Moreover, to validate the consistency of sCCA results regardless of window size for computation of dFCs, we also used dFC matrices obtained by FLS methods to measure canonical correlations between brain dynamics and clinical variables with other parameters the same as the main sCCA analysis. The sCCA findings of using FLS to obtain dFCs and using PCA for feature dimension reduction yielded similar results as the main analysis (Fig. S6 and S7).

*Calculate sparse canonical correlation*

sCCA is a multivariate correlation procedure that seeks maximal linear combinations of multidimensional variables in both sets, with regulation to achieve sparsity (Witten, Tibshirani, & Hastie, 2009). In brief, given two matrices, X*_n×p_* and Y*_n×q_*, in which *p* and *q* are the number of features (e.g., brain imaging feature and clinical scores, respectively), and *n* is the number of participants. sCCA involves finding u and v, which are loading vectors, that maximize cor (Xu,Yv). Since both *L*^1^-norm and *L*^2^-norm are used, this is an elastic net regularization that combines the LASSO and ridge penalties. We tuned the *L*^1^ regulation parameters for dFC and clinical features (behavior and cognition, respectively). The range of sparsity ranged from 0 to 1 in the PMA package (Witten et al., 2009) in R, where 1 indicates preserving all the features (lowest sparsity level) and 0 indicates the least number of features (highest sparsity level). The grid search method with increment was used to determine the combination of parameters that would achieve the highest correlation of the first canonical variate across 300 randomly resampled samples. The best sparsity parameters for sparse canonical correlation between dFC and behaviors or cognitive functions are shown in Fig. S4. The best sparsity parameters were applied to compute the canonical correlations.

*Permutation test*

To assess the statistical significance of each canonical variate, a permutation test was used to create the null distribution of canonical correlations. First, we held the dFC features constant, and then shuffled the rows of clinical scores to break the linkage of participants’ brain features and clinical features. Then, we performed sCCA using the same sparsity parameters as before to generate a null distribution of correlations after permuting 1000 times. Because permutation could induce arbitrary axis rotation, which changes the order of canonical variates, we matched the canonical variates of permuted data matrices with the ones derived from the original data matrix by comparing the clinical loadings (Misic et al., 2016). The *P* value was estimated as the percentage of null correlations that exceeded the primary sCCA correlations estimated on the original data. The false discovery rate (FDR) correction was used to correct the *P* value across the selected canonical variates, which preserve 85% of covariance explained.

*Resampling procedure*

To estimate mean and standard error for canonical correlation coefficients and confidence intervals (CI) for variable loadings, we applied a bootstrapping procedure, resampling our data with replacements for 1000 times. Similar to the permutation procedure, we matched the canonical variates from resampled matrices to the original one to obtain a set of comparable decompositions (Chai et al., 2017). Features whose 99% (for significant brain imaging features, Fig. 3A and B) and 95% (for clinical features, Fig. S8) CI of loading value did not cross zero were considered as significant, suggesting that they were stable across different sampling cohorts. To validate if these stable features significantly related with their respective linear combined variates (canonical variates), we next calculated the Pearson’s correlation between each original variable and the corresponding canonical variate.

*Interpretation of canonical variates*

Finally, to understand the meaning of each significant brain imaging canonical variate and make visualization, we calculated the sum of absolute loading values for within- and between-networks dFC features.

**1.4 Mediation analysis**

Pearson correlations were computed to identify significant relationships between pairs of dimension scores (linear combination score) of cognition and behavior. The correlation analysis revealed that the inhibition and flexibility dimension score significantly correlated with inattention/hyperactivity dimension score (*r* = -0.429, *P* < 0.001), while the other three pairs didn’t show a significant correlation. Therefore, we inferred that cognitive functions involving inhibitory control and cognitive flexibility mediate the relationship between dFC and inattention/hyperactivity. For brain imaging characteristics, we treated dFCs related to both cognition and behavior in the mediation analyses. We selected overlapping dFCs that significantly contributed to both the cognition dimension of inhibition and flexibility as well as to the behavior dimension of inattention/hyperactivity for the mediation analysis. Then we calculated the mean value of all dFCs positively or negatively correlated with the cognition dimension score to represent two types of overlapping dFCs (named positive links and negative links, respectively). Subsequently, each type of shared dFC was used for mediation analysis to test our assumption that cognitive function could mediate the relationship between brain functional dynamics and behaviors. Covariates used in this model included age and sex.

Several paths between variables were estimated in the mediation analyses, including the total effect, direct effect, and indirect effect. For an independent variable X, mediator M, and a dependent variable Y, the total effect of X on Y consists of the direct effect of X on Y after controlling for M (path c’) and the indirect effect of X on Y through M (e.g. path X→M and M→Y, also known as path a × b). The mediation effect is significant when the simulated 95% CI does not include zero. The proportion of mediation indicates the percentage of indirect effects explained for the total effect of X on Y. The default simulation type is the quasi-Bayesian Monte Carlo method (1000 times) (Tingley, Yamamoto, Hirose, Keele, & Imai, 2014). The two-tailed α was set at 0.05.

**2. Supplementary Results**

**2.1 The diagnosis*sex interactions on behavioral and cognitive scores.**

We tested the main effect of diagnosis and sex, as well as the diagnosis*sex interactions on the cognitive or behavioral ratings (Table S2). The results showed that there was a significant interaction effect of diagnosis*sex only on the anxiety score, no significant interaction effect of diagnosis*sex was observed on other cognitive or behavioral scores.

We have also tested group differences in cognitive or behavioral scores for boys and girls, as well as sex differences in cognitive or behavioral scores of TDC and ADHD, respectively (Table S3). As shown in Table S3, for boys and girls, the diagnostic group differences were similar in most cognitive or behavioral scores. While for boys the diagnostic group differences were more prominent in most clinical scores than girls, probably because the sample size of boys (n = 106) was larger than girls (n =16). We also found ADHD girls had significantly higher anxiety score and social withdrawn score than typically developing girls, but this trend was not significant in boys. As for the sex differences in cognitive or behavioral scores of TDC and ADHD, we only observed girls with ADHD showed significantly higher anxiety score than boys with ADHD. Together with the finding of significant diagnosis*sex interaction effect on the anxiety score of all subjects, we found ADHD girls had higher anxiety level than ADHD boys and typically developing girls. Therefore, sex may have a different effect on anxiety level of TDC and ADHD, but have no main effect or diagnosis*sex interaction effect on other cognitive or behavioral scores.

**2.2 Canonical correlation patterns between dFC and clinical assessments**

*Linked dimensions of canonical variates*

We performed sCCA to link dFC with behavioral measures and cognitive function scores, respectively. Specifically, for dFC-behavior sCCA analysis, we selected the first 12 pairs of canonical variates for further analysis, based on their accumulated covariance explained more than 85% (Fig. S5A). Of these 12 canonical variates, two were significant using permutation tests with FDR correction (*r*_mean_ = 0.857, *P* < 0.001, *P*_FDR_ < 0.001; *r*_mean_ = 0.836, *P* = 0.007, *P*_FDR_ = 0.042, respectively), while another one canonical variate was nominally significant (*r*_mean_ = 0.818, *P* = 0.042, *P*_FDR_ = 0.168) (Fig. S5B).

For the dFC-cognition sCCA analysis, the first 14 pairs of canonical variates were selected for further analysis based on the accumulated covariance explained (Fig. S5C). Of these 14 canonical variates, six were significant after permutation tests, in which two survived the FDR correction (*r*_mean_ = 0.864, *P* < 0.001, *P*_FDR_ < 0.001; *r*_mean_ = 0.855, *P* < 0.001, *P*_FDR_ < 0.001, respectively), while the other four were not significant after the FDR correction (Fig. S5D). Notably, these results were similar to the results of using PCA for feature dimension reduction (Fig. S6) or using the FLS method to calculate dFC matrices (Fig. S7), indicating that the multivariate correlations were robust regardless of different methodological choices.

Each canonical variate represented a characteristic pattern that relates a weighted set of dFCs to a weighted set of clinical scores. Inspection of the clinical scores with the heaviest weights for each dimension guided interpretation regarding their content (Fig. 2). For example, in the analysis of behaviors, attention problems, hyperactivity index, and study problems contributed most to the first significant dimension, we named this dimension as “inattention/hyperactivity” (Fig. 2B); while psychosomatic manifestation was the most heavily weighted clinical feature in the second significant behavior dimension, we named this dimension as “somatization” (Fig. 2E). For the analysis of cognitive functions, Stroop Test and WCST scores contributed most to the first significant dimension, we summarized this cognition dimension as “inhibition and flexibility” (Fig. 2H). As Fluency Test and Visual Memory scores weighted most heavily in the second significant cognition dimension, we called this dimension “fluency and memory” (Fig. 2K).

**3. Supplementary Tables**

**Table S1.** Demographic and clinical characteristics of children with attention-deficit/hyperactivity disorder (ADHD) and typically developing children (TDC).

| Clinical scores | TDC  (n = 59) | ADHD  (n = 63) | χ^2^/t value | *P* value |  |
| --- | --- | --- | --- | --- | --- |
| Sex (number) | 49M, 10F | 57M, 6F | 1.474 | 0.225 |  |
| Age (years) | 10.6 ± 1.9 | 10.3±2.3 | 0.872 | 0.385 |  |
| **Corners' Parent Rating Scale** | | | | |  |
| Conduct problems | 5.276±4.797 | 12.017±7.604 | -6.741 | 0.000** |  |
| Study problems | 2.897±2.532 | 7.262±2.834 | -4.366 | 0.000** |  |
| Psychosomatic problems | 0.517±1.096 | 1.066±1.276 | -0.548 | 0.013** |  |
| Impulsive-hyperactive | 2.138±1.951 | 5.590±3.063 | -3.452 | 0.000** |  |
| Anxiety | 1.534±1.570 | 1.656±1.769 | -0.121 | 0.694 |  |
| Hyperactivity index | 4.983±4.579 | 13.233±5.998 | -8.251 | 0.000** |  |
| **Achenbach Children Behavior Checklist** | | | | |  |
| Social withdrawn | 3.298±3.257 | 4.131±3.354 | -0.833 | 0.174 |  |
| Somatic complaints | 1.228±1.881 | 1.328±1.690 | -0.100 | 0.762 |  |
| Anxious/Depressed | 2.764±3.344 | 5.803±4.932 | -3.040 | 0.000** |  |
| Uncommunicative | 2.754±2.481 | 5.180±2.958 | -2.426 | 0.000** |  |
| Thought problems | 0.509±0.928 | 2.068±2.399 | -1.559 | 0.000** |  |
| Attention problems | 3.719±3.161 | 9.517±3.553 | -5.797 | 0.000** |  |
| Delinquent problems | 2.054±1.773 | 5.117±3.385 | -3.063 | 0.000** |  |
| Aggressive behaviors | 5.946±4.777 | 14.213±8.118 | -8.267 | 0.000** |  |
| Internalizing | 6.406±7.783 | 11.324±8.130 | -4.917 | 0.015** |  |
| Externalizing | 7.927±6.158 | 19.571±11.043 | -11.644 | 0.000** |  |
| **Visual Memory** | | | | |  |
| Immediate score | 19.140±4.962 | 17.242±4.693 | 1.898 | 0.034** |  |
| Delayed score | 18.696±4.628 | 16.344±4.468 | 2.352 | 0.006** |  |
| **Stroop Test** |  |  |  |  |  |
| Stroop-C No. right | 110.576±1.704 | 108.794±3.686 | 1.783 | 0.001** |  |
| Stroop-C No. error | 1.153±1.495 | 2.937±3.454 | -1.784 | 0.000** |  |
| Stroop-C No. correction | 0.797±1.156 | 1.095±1.201 | -0.299 | 0.165 |  |
| Stroop-C Total score | 111.390±1.034 | 110.048±3.353 | 1.342 | 0.004** |  |
| Stroop-C Total time | 74.017±18.677 | 94.190±32.772 | -20.174 | 0.000* |  |
| Stroop-CW No. right | 104.814±6.277 | 96.557±10.264 | 8.256 | 0.000* |  |
| Stroop-CW No. error | 6.119±5.623 | 15.016±10.380 | -8.898 | 0.000* |  |
| Stroop-CW No. correction | 2.847±2.310 | 6.902±5.075 | -4.054 | 0.000* |  |
| Stroop-CW Total score | 105.966±13.770 | 103.459±9.239 | 2.507 | 0.242 |  |
| Stroop-CW Total time | 175.373±44.176 | 267.033±97.386 | -91.660 | 0.000* |  |
| **Fluency Test** | | | | |  |
| Word Total score | 18.525±4.772 | 17.667±6.912 | 0.859 | 0.429 |  |
| Word No. error | 0.085±0.337 | 0.016±0.126 | 0.069 | 0.133 |  |
| Word No. perseverative | 0.288±0.589 | 0.762±1.266 | -0.474 | 0.010* |  |
| Word No. correct | 17.949±5.107 | 16.889±7.005 | 1.060 | 0.344 |  |
| Ideational No. correct | 12.458±5.781 | 12.127±7.124 | 0.331 | 0.780 |  |
| non-VFT No. correct | 15.000±7.826 | 12.635±6.769 | 2.365 | 0.076 |  |
| **Wisconsin Card Sorting Test** | | | | |  |
| Total correct matches | 35.255±6.407 | 29.806±10.114 | 5.448 | 0.001* |  |
| Total errors | 9.600±7.788 | 16.242±11.881 | -6.642 | 0.001* |  |
| Perseverative errors | 2.055±2.606 | 4.952±5.427 | -2.897 | 0.000* |  |
| Noperserverative errors | 7.545±5.865 | 11.290±7.502 | -3.745 | 0.004* |  |
| Categories completed | 5.291±1.595 | 4.306±1.861 | 0.984 | 0.003* |  |
| **Digital span (forward)** | 8.254±1.708 | 7.286±2.059 | 0.969 | 0.006* |  |

Values were given as mean±SD. *P* value of sex was obtained by chi-square test and *P* values of other measures were obtained by two-sample *t*-test. * indicates *P* < 0.05.

Abbreviations: TDC, typical developing children; ADHD, Attention-Deficit/Hyperactivity Disorder; M, male; F, female; Stroop-C/CW, Stroop Color/Color-Word Test; No., number of; VFT, Verbal Fluency Test.

**Table S2.** The main effects of diagnosis and sex, as well as the diagnosis*sex interactions on the cognitive or behavioral ratings.

| Clinical scores | Group | |  | Sex | |  | Group*sex | |
| --- | --- | --- | --- | --- | --- | --- | --- | --- |
|  | *F* value | *P* value |  | *F* value | *P* value |  | *F* value | *P* value |
| **Corners' Parent Rating Scale** | | | | | |  |  |  |
| Conduct problems | 15.592 | 0.000* |  | 0.432 | 0.512 |  | 0.147 | 0.702 |
| Study problems | 42.984 | 0.000* |  | 0.019 | 0.889 |  | 0.975 | 0.325 |
| Psychosomatic problems | 1.491 | 0.225 |  | 1.114 | 0.293 |  | 0.511 | 0.476 |
| Impulsive-hyperactive | 18.395 | 0.000* |  | 0.129 | 0.720 |  | 0.572 | 0.451 |
| Anxiety | 4.504 | 0.036* |  | 3.031 | 0.084 |  | 5.600 | 0.020* |
| Hyperactivity index | 27.985 | 0.000* |  | 0.007 | 0.935 |  | 0.003 | 0.953 |
| **Achenbach Children Behavior Checklist** | | | | | |  |  |  |
| Social withdrawn | 5.674 | 0.019* |  | 1.484 | 0.226 |  | 3.554 | 0.062 |
| Somatic complaints | 0.628 | 0.430 |  | 0.499 | 0.482 |  | 2.092 | 0.151 |
| Anxious/Depressed | 6.132 | 0.015* |  | 0.590 | 0.444 |  | 0.016 | 0.899 |
| Uncommunicative | 16.457 | 0.000* |  | 1.988 | 0.161 |  | 1.032 | 0.312 |
| Thought problems | 10.778 | 0.001* |  | 0.488 | 0.486 |  | 0.060 | 0.808 |
| Attention problems | 34.625 | 0.000* |  | 0.185 | 0.668 |  | 0.202 | 0.654 |
| Delinquent problems | 17.993 | 0.000* |  | 0.279 | 0.599 |  | 0.128 | 0.721 |
| Aggressive behaviors | 16.779 | 0.000* |  | 0.000 | 0.999 |  | 0.178 | 0.674 |
| Internalizing | 4.457 | 0.039* |  | 1.738 | 0.192 |  | 0.030 | 0.864 |
| Externalizing | 18.823 | 0.000* |  | 0.007 | 0.935 |  | 0.026 | 0.871 |
| **Visual Memory** | | | | | |  |  |  |
| Immediate score | 1.590 | 0.210 |  | 4.027 | 0.050 |  | 0.001 | 0.980 |
| Delayed score | 4.308 | 0.040* |  | 3.187 | 0.077 |  | 0.210 | 0.645 |
| **Stroop Test** |  |  |  |  |  |  |  |  |
| Stroop-C No. right | 5.918 | 0.017* |  | 0.012 | 0.914 |  | 0.086 | 0.770 |
| Stroop-C No. error | 5.267 | 0.024* |  | 0.001 | 0.970 |  | 0.013 | 0.909 |
| Stroop-C No. correction | 1.083 | 0.300 |  | 1.384 | 0.242 |  | 0.083 | 0.773 |
| Stroop-C Total score | 4.911 | 0.029* |  | 0.665 | 0.416 |  | 0.100 | 0.753 |
| Stroop-C Total time | 8.835 | 0.004* |  | 0.333 | 0.565 |  | 0.181 | 0.671 |
| Stroop-CW No. right | 6.995 | 0.009* |  | 0.027 | 0.870 |  | 1.294 | 0.258 |
| Stroop-CW No. error | 9.671 | 0.002* |  | 0.181 | 0.672 |  | 0.870 | 0.353 |
| Stroop-CW No. correction | 6.347 | 0.013* |  | 2.103 | 0.150 |  | 2.275 | 0.134 |
| Stroop-CW Total score | 0.588 | 0.445 |  | 0.003 | 0.959 |  | 0.000 | 0.993 |
| Stroop-CW Total time | 15.821 | 0.000* |  | 0.514 | 0.475 |  | 0.178 | 0.674 |
| **Fluency Test** | | | | | |  |  |  |
| Word Total score | 0.017 | 0.896 |  | 0.214 | 0.645 |  | 0.329 | 0.568 |
| Word No. error | 1.380 | 0.242 |  | 0.000 | 0.995 |  | 0.066 | 0.798 |
| Word No. perseverative | 1.998 | 0.160 |  | 0.510 | 0.477 |  | 0.109 | 0.741 |
| Word No. correct | 0.056 | 0.814 |  | 0.068 | 0.795 |  | 0.300 | 0.585 |
| Ideational No. correct | 0.085 | 0.771 |  | 0.037 | 0.848 |  | 0.028 | 0.867 |
| non-VFT No. correct | 0.127 | 0.772 |  | 0.162 | 0.688 |  | 1.324 | 0.252 |
| **Wisconsin Card Sorting Test** | | | | | |  |  |  |
| Total correct matches | 5.848 | 0.017* |  | 0.004 | 0.950 |  | 0.088 | 0.768 |
| Total errors | 6.932 | 0.010* |  | 0.011 | 0.916 |  | 0.243 | 0.623 |
| Perseverative errors | 4.889 | 0.029* |  | 0.242 | 0.624 |  | 0.024 | 0.877 |
| Noperserverative errors | 6.444 | 0.012* |  | 0.025 | 0.876 |  | 0.705 | 0.403 |
| Categories completed | 4.790 | 0.031* |  | 0.147 | 0.702 |  | 0.083 | 0.773 |
| **Digital span (forward)** | 6.924 | 0.010* |  | 0.065 | 0.799 |  | 1.096 | 0.297 |

* indicates *P* < 0.05.

Abbreviations: Stroop-C/CW, Stroop Color/Color-Word Test; No., number of; VFT, Verbal Fluency Test.

**Table S3.** Diagnostic group differences of cognitive or behavioral scores for boys and girls, as well as sex differences of cognitive or behavioral scores of typically developing children (TDC) and children with attention-deficit/hyperactivity disorder (ADHD).

| Clinical scores | boys (TDC vs ADHD) | |  | girls (TDC vs ADHD) | |  | TDC (girls vs boys) | |  | ADHD (girls vs boys) | |
| --- | --- | --- | --- | --- | --- | --- | --- | --- | --- | --- | --- |
|  | *T* value | *P* value |  | *T* value | *P* value |  | *T* value | *P* value |  | *T* value | *P* value |
| **Corners' Parent Rating Scale** | | | | | |  |  |  |  |  |  |
| Conduct problems | -5.247 | 0.000* |  | -2.332 | 0.036* |  | 0.305 | 0.762 |  | 0.544 | 0.588 |
| Study problems | -7.486 | 0.000* |  | -6.419 | 0.000* |  | -0.956 | 0.343 |  | 0.517 | 0.607 |
| Psychosomatic problems | -2.778 | 0.007* |  | -0.233 | 0.819 |  | 1.550 | 0.127 |  | 0.203 | 0.840 |
| Impulsive-hyperactive | -7.160 | 0.000* |  | -1.727 | 0.106 |  | 1.184 | 0.241 |  | -0.215 | 0.831 |
| Anxiety | 0.345 | 0.730 |  | -2.236 | 0.042* |  | -0.516 | 0.608 |  | 2.557 | 0.013* |
| Hyperactivity index | -7.538 | 0.000* |  | -3.552 | 0.004* |  | -0.138 | 0.891 |  | -0.013 | 0.990 |
| **Achenbach Children Behavior Checklist** | | | | | |  |  |  |  |  |  |
| Social withdrawn | -0.680 | 0.498 |  | -2.636 | 0.020* |  | -0.529 | 0.599 |  | 1.998 | 0.050 |
| Somatic complaints | -0.914 | 0.363 |  | 1.164 | 0.264 |  | 1.639 | 0.107 |  | -0.497 | 0.621 |
| Anxious/Depressed | -3.567 | 0.001* |  | -1.703 | 0.112 |  | 0.884 | 0.381 |  | 0.362 | 0.719 |
| Uncommunicative | -4.224 | 0.000* |  | -2.766 | 0.015* |  | 0.342 | 0.734 |  | 1.455 | 0.151 |
| Thought problems | -4.139 | 0.000* |  | -2.127 | 0.052 |  | 0.714 | 0.478 |  | 0.462 | 0.645 |
| Attention problems | -8.679 | 0.000* |  | -3.218 | 0.006* |  | 0.747 | 0.458 |  | -0.012 | 0.990 |
| Delinquent problems | -5.287 | 0.000* |  | -2.759 | 0.015* |  | -1.091 | 0.280 |  | -0.088 | 0.930 |
| Aggressive behaviors | -6.042 | 0.000* |  | -2.849 | 0.013* |  | 0.474 | 0.637 |  | -0.225 | 0.823 |
| Internalizing | -2.160 | 0.035* |  | -2.190 | 0.053 |  | 0.885 | 0.383 |  | 0.975 | 0.337 |
| Externalizing | -6.011 | 0.000* |  | -3.187 | 0.007* |  | 0.097 | 0.923 |  | -0.121 | 0.904 |
| **Visual Memory** | | | | | |  |  |  |  |  |  |
| Immediate score | 1.723 | 0.088 |  | 1.092 | 0.293 |  | 1.534 | 0.131 |  | 1.340 | 0.185 |
| Delayed score | 2.167 | 0.033* |  | 1.844 | 0.086 |  | 1.770 | 0.082 |  | 0.858 | 0.394 |
| **Stroop Test** |  |  |  |  |  |  |  |  |  |  |  |
| Stroop-C No. right | 2.966 | 0.004* |  | 1.765 | 0.099 |  | 0.250 | 0.804 |  | -0.204 | 0.839 |
| Stroop-C No. error | -3.305 | 0.001* |  | -1.634 | 0.124 |  | 0.109 | 0.913 |  | -0.076 | 0.939 |
| Stroop-C No. correction | -1.057 | 0.293 |  | -0.756 | 0.462 |  | -1.194 | 0.237 |  | -0.558 | 0.579 |
| Stroop-C Total score | 2.605 | 0.011* |  | 1.925 | 0.075 |  | -0.972 | 0.335 |  | -0.546 | 0.587 |
| Stroop-C Total time | -3.695 | 0.000* |  | -1.564 | 0.140 |  | -1.158 | 0.252 |  | -0.080 | 0.937 |
| Stroop-CW No. right | 5.355 | 0.000* |  | 0.769 | 0.454 |  | -1.059 | 0.294 |  | 0.695 | 0.490 |
| Stroop-CW No. error | -5.615 | 0.000* |  | -1.289 | 0.218 |  | 0.602 | 0.549 |  | -0.705 | 0.483 |
| Stroop-CW No. correction | -5.670 | 0.000* |  | -0.536 | 0.600 |  | 0.078 | 0.938 |  | -1.490 | 0.142 |
| Stroop-CW Total score | 1.039 | 0.301 |  | 0.803 | 0.435 |  | -0.041 | 0.967 |  | -0.035 | 0.972 |
| Stroop-CW Total time | -5.996 | 0.000* |  | -2.619 | 0.020* |  | -0.403 | 0.688 |  | -0.572 | 0.570 |
| **Fluency Test** | | | | | |  |  |  |  |  |  |
| Word Total score | 0.958 | 0.340 |  | -0.388 | 0.704 |  | -1.037 | 0.304 |  | 0.062 | 0.951 |
| Word No. error | 1.300 | 0.196 |  | 0.764 | 0.458 |  | 0.156 | 0.877 |  | -0.322 | 0.748 |
| Word No. perseverative | -2.371 | 0.020* |  | -0.963 | 0.352 |  | -0.516 | 0.608 |  | -0.529 | 0.598 |
| Word No. correct | 1.069 | 0.287 |  | -0.259 | 0.800 |  | -0.778 | 0.440 |  | 0.162 | 0.872 |
| Ideational No. correct | 0.172 | 0.864 |  | 0.296 | 0.771 |  | 0.323 | 0.748 |  | 0.014 | 0.989 |
| non-VFT No. correct | 2.073 | 0.041* |  | -0.574 | 0.575 |  | -1.156 | 0.252 |  | 0.516 | 0.608 |
| **Wisconsin Card Sorting Test** | | | | | |  |  |  |  |  |  |
| Total correct matches | 3.000 | 0.003* |  | 2.011 | 0.067 |  | 0.235 | 0.815 |  | -0.204 | 0.839 |
| Total errors | -3.039 | 0.003* |  | -2.161 | 0.052 |  | -0.380 | 0.705 |  | 0.343 | 0.733 |
| Perseverative errors | -3.395 | 0.001* |  | -1.246 | 0.237 |  | 0.814 | 0.419 |  | 0.180 | 0.858 |
| Noperserverative errors | -2.401 | 0.018* |  | -2.511 | 0.027* |  | -0.869 | 0.389 |  | 0.413 | 0.681 |
| Categories completed | 2.738 | 0.007* |  | 1.429 | 0.179 |  | -0.078 | 0.938 |  | -0.422 | 0.675 |
| **Digital span (forward)** | 2.309 | 0.023* |  | 1.654 | 0.120 |  | 0.699 | 0.487 |  | -0.772 | 0.443 |

* indicates *P* < 0.05.

Abbreviations: TDC, typical developing children; ADHD, Attention-Deficit/Hyperactivity Disorder; Stroop-C/CW, Stroop Color/Color-Word Test; No., number of; VFT, Verbal Fluency Test.

**Table S4.** Differences in clinical scores between different sexes and correlations between age and clinical scores in all participants.

| Clinical scores | Differences between sexes | |  | Correlations with age | |
| --- | --- | --- | --- | --- | --- |
|  | *t* value | *P* value |  | *r* value | *P* value |
| **Corners’ Parent Rating Scale** | | | | | |
| Conduct problems | 0.174 | 0.862 |  | -0.130 | 0.160 |
| Study problems | 1.021 | 0.309 |  | 0.072 | 0.439 |
| Psychosomatic problems | -0.932 | 0.353 |  | 0.032 | 0.733 |
| Impulsive-hyperactive | 0.218 | 0.828 |  | -0.171 | 0.063 |
| Anxiety | -1.202 | 0.232 |  | 0.080 | 0.385 |
| Hyperactivity index | 0.969 | 0.334 |  | -0.124 | 0.181 |
| **Achenbach Children Behavior Checklist** | | | | | |
| Social withdrawn | -0.674 | 0.502 |  | 0.001 | 0.988 |
| Somatic complaints | -0.987 | 0.326 |  | 0.033 | 0.727 |
| Anxious/Depressed | -0.402 | 0.688 |  | -0.022 | 0.815 |
| Uncommunicative | -0.617 | 0.539 |  | -0.025 | 0.788 |
| Thought problems | -0.158 | 0.874 |  | 0.138 | 0.138 |
| Attention problems | 0.367 | 0.714 |  | -0.027 | 0.776 |
| Delinquent problems | 1.141 | 0.256 |  | -0.087 | 0.356 |
| Aggressive behaviors | 0.582 | 0.562 |  | -0.167 | 0.072 |
| Internalizing | -1.030 | 0.307 |  | -0.062 | 0.620 |
| Externalizing | 0.666 | 0.507 |  | -0.240 | 0.014 |
| **Visual Memory** | | | | | |
| Immediate score | -2.266 | 0.025* |  | 0.305 | 0.001* |
| Delayed score | -2.191 | 0.030* |  | 0.250 | 0.007* |
| **Stroop Test** |  |  |  |  |  |
| Stroop-C No. right | -0.310 | 0.757 |  | 0.178 | 0.050 |
| Stroop-C No. error | 0.395 | 0.693 |  | -0.166 | 0.068 |
| Stroop-C No. correction | 1.413 | 0.160 |  | -0.079 | 0.385 |
| Stroop-C total score | 0.427 | 0.670 |  | 0.138 | 0.131 |
| Stroop-C total time | 1.068 | 0.288 |  | -0.490 | 0.001* |
| Stroop-CW No. right | -0.428 | 0.669 |  | 0.166 | 0.070 |
| Stroop-CW No. error | 0.743 | 0.459 |  | -0.173 | 0.059 |
| Stroop-CW No. correction | 1.553 | 0.123 |  | -0.096 | 0.299 |
| Stroop-CW total score | -0.067 | 0.947 |  | 0.111 | 0.226 |
| Stroop-CW total time | 1.146 | 0.254 |  | -0.465 | 0.001* |
| **Fluency Test** | | | | | |
| Word Total score | 0.507 | 0.613 |  | 0.360 | 0.001* |
| Word No. error | -0.226 | 0.822 |  | 0.084 | 0.355 |
| Word No. perseverative | 0.924 | 0.357 |  | -0.094 | 0.304 |
| Word No. correct | 0.279 | 0.781 |  | 0.341 | 0.000* |
| Ideational No. correct | -0.264 | 0.792 |  | 0.153 | 0.092 |
| non-VFT No. correct | 0.452 | 0.652 |  | 0.183 | 0.043* |
| **Wisconsin Card Sorting Test** | | | | | |
| Total correct matches | -0.217 | 0.829 |  | 0.184 | 0.047 |
| Total errors | 0.204 | 0.838 |  | -0.232 | 0.012* |
| Perseverative errors | -0.233 | 0.816 |  | -0.199 | 0.032* |
| Non-perseverative errors | 0.462 | 0.645 |  | -0.223 | 0.015* |
| Categories completed | 0.121 | 0.904 |  | 0.166 | 0.073 |
| **Digital Span (forward)** | -0.265 | 0.792 |  | 0.165 | 0.069 |

The *t* values and *P* values of differences between sexes are obtained by two-sample *t*-test, and *t* > 0 represents the clinical score of male > female. The *r* values are the Pearson correlation coefficients between age and clinical scores across all participants. The * indicates uncorrected *P* < 0.05. Abbreviations: Stroop-C/CW, Stroop Color/Color-Word Test; No., number of; VFT, non-Verbal Fluency Test.

**Table S5.** The information about the 227 brain regions of the Power atlas used in our study.

| ROI | Network | Location | Brain region | Abbreviation | MNI coordinate | | |
| --- | --- | --- | --- | --- | --- | --- | --- |
|  |  |  |  |  | X | Y | Z |
| 1 | SMN | L | Precuneus | Pcu | -7 | -52 | 61 |
| 2 | SMN | L | Cingulate Gyrus | CG | -14 | -18 | 40 |
| 3 | SMN | L | Paracentral Lobule | PCL | 0 | -15 | 47 |
| 4 | SMN | R | Cingulate Gyrus | CG | 10 | -2 | 45 |
| 5 | SMN | L | Medial Frontal Gyrus | MFG | -7 | -21 | 65 |
| 6 | SMN | L | Paracentral Lobule | PCL | -7 | -33 | 72 |
| 7 | SMN | R | Paracentral Lobule | PCL | 13 | -33 | 75 |
| 8 | SMN | L | Postcentral Gyrus | PCG | -54 | -23 | 43 |
| 9 | SMN | R | Precentral Gyrus | PreG | 29 | -17 | 71 |
| 10 | SMN | R | Postcentral Gyrus | PCG | 10 | -46 | 73 |
| 11 | SMN | L | Postcentral Gyrus | PCG | -23 | -30 | 72 |
| 12 | SMN | L | Postcentral Gyrus | PCG | -40 | -19 | 54 |
| 13 | SMN | R | Sub-Gyral | SG | 29 | -39 | 59 |
| 14 | SMN | R | Postcentral Gyrus | PCG | 50 | -20 | 42 |
| 15 | SMN | L | Postcentral Gyrus | PCG | -38 | -27 | 69 |
| 16 | SMN | R | Paracentral Lobule | PCL | 20 | -29 | 60 |
| 17 | SMN | R | Precentral Gyrus | PreG | 44 | -8 | 57 |
| 18 | SMN | L | Sub-Gyral | SG | -29 | -43 | 61 |
| 19 | SMN | R | Medial Frontal Gyrus | MFG | 10 | -17 | 74 |
| 20 | SMN | R | Superior Parietal Lobule | SPL | 22 | -42 | 69 |
| 21 | SMN | L | Inferior Parietal Lobule | IPL | -45 | -32 | 47 |
| 22 | SMN | L | Postcentral Gyrus | PCG | -21 | -31 | 61 |
| 23 | SMN | L | Precentral Gyrus | PreG | -13 | -17 | 75 |
| 24 | SMN | R | Postcentral Gyrus | PCG | 42 | -20 | 55 |
| 25 | SMN | L | Precentral Gyrus | PreG | -38 | -15 | 69 |
| 26 | SMN | L | Postcentral Gyrus | PCG | -16 | -46 | 73 |
| 27 | SMN | L | Paracentral Lobule | PCL | 2 | -28 | 60 |
| 28 | SMN | R | Medial Frontal Gyrus | MFG | 3 | -17 | 58 |
| 29 | SMN | R | Postcentral Gyrus | PCG | 38 | -17 | 45 |
| 30 | SMN | R | Inferior Parietal Lobule | IPL | 47 | -30 | 49 |
| 31 | SMN | L | Precentral Gyrus | PreG | -49 | -11 | 35 |
| 32 | SMN | R | Claustrum | Cla | 36 | -9 | 14 |
| 33 | SMN | R | Precentral Gyrus | PreG | 51 | -6 | 32 |
| 34 | SMN | L | Postcentral Gyrus | PCG | -53 | -10 | 24 |
| 35 | SMN | R | Postcentral Gyrus | PCG | 66 | -8 | 25 |
| 36 | CON | L | Medial Frontal Gyrus | MFG | -3 | 2 | 53 |
| 37 | CON | R | Inferior Parietal Lobule | IPL | 54 | -28 | 34 |
| 38 | CON | R | Superior Frontal Gyrus | SFG | 19 | -8 | 64 |
| 39 | CON | L | Superior Frontal Gyrus | SFG | -16 | -5 | 71 |
| 40 | CON | L | Cingulate Gyrus | CG | -10 | -2 | 42 |
| 41 | CON | R | Claustrum | Cla | 37 | 1 | -4 |
| 42 | CON | R | Superior Frontal Gyrus | SFG | 13 | -1 | 70 |
| 43 | CON | R | Medial Frontal Gyrus | MFG | 7 | 8 | 51 |
| 44 | CON | L | Insula | Insula | -45 | 0 | 9 |
| 45 | CON | R | Insula | Insula | 49 | 8 | -1 |
| 46 | CON | L | Claustrum | Cla | -34 | 3 | 4 |
| 47 | CON | L | Superior Temporal Gyrus | STG | -51 | 8 | -2 |
| 48 | CON | L | Cingulate Gyrus | CG | -5 | 18 | 34 |
| 49 | CON | R | Claustrum | Cla | 36 | 10 | 1 |
| 50 | AUD | R | Insula | Insula | 32 | -26 | 13 |
| 51 | AUD | R | Superior Temporal Gyrus | STG | 65 | -33 | 20 |
| 52 | AUD | R | Transverse Temporal Gyrus | TTG | 58 | -16 | 7 |
| 53 | AUD | L | Superior Temporal Gyrus | STG | -38 | -33 | 17 |
| 54 | AUD | L | Superior Temporal Gyrus | STG | -60 | -25 | 14 |
| 55 | AUD | L | Superior Temporal Gyrus | STG | -49 | -26 | 5 |
| 56 | AUD | R | Insula | Insula | 43 | -23 | 20 |
| 57 | AUD | L | Insula | Insula | -50 | -34 | 26 |
| 58 | AUD | L | Postcentral Gyrus | PCG | -53 | -22 | 23 |
| 59 | AUD | L | Precentral Gyrus | PreG | -55 | -9 | 12 |
| 60 | AUD | R | Precentral Gyrus | PreG | 56 | -5 | 13 |
| 61 | AUD | R | Postcentral Gyrus | PCG | 59 | -17 | 29 |
| 62 | AUD | L | Insula | Insula | -30 | -27 | 12 |
| 63 | DMN | L | Middle Temporal Gyrus | MTG | -41 | -75 | 26 |
| 64 | DMN | R | Medial Frontal Gyrus | MFG | 6 | 67 | -4 |
| 65 | DMN | R | Medial Frontal Gyrus | MFG | 8 | 48 | -15 |
| 66 | DMN | L | Parahippocampal Gyrus | PHG | -13 | -40 | 1 |
| 67 | DMN | L | Medial Frontal Gyrus | MFG | -18 | 63 | -9 |
| 68 | DMN | L | Superior Temporal Gyrus | STG | -46 | -61 | 21 |
| 69 | DMN | R | Middle Temporal Gyrus | MTG | 43 | -72 | 28 |
| 70 | DMN | L | Superior Temporal Gyrus | STG | -44 | 12 | -34 |
| 71 | DMN | R | Superior Temporal Gyrus | STG | 46 | 16 | -30 |
| 72 | DMN | L | Middle Temporal Gyrus | MTG | -68 | -23 | -16 |
| 73 | DMN | L | Middle Temporal Gyrus | MTG | -44 | -65 | 35 |
| 74 | DMN | L | Precuneus | Pcu | -39 | -75 | 44 |
| 75 | DMN | L | Posterior Cingulate | PC | -7 | -55 | 27 |
| 76 | DMN | R | Precuneus | Pcu | 6 | -59 | 35 |
| 77 | DMN | L | Posterior Cingulate | PC | -11 | -56 | 16 |
| 78 | DMN | L | Posterior Cingulate | PC | -3 | -49 | 13 |
| 79 | DMN | R | Cingulate Gyrus | CG | 8 | -48 | 31 |
| 80 | DMN | R | Precuneus | Pcu | 15 | -63 | 26 |
| 81 | DMN | L | Cingulate Gyrus | CG | -2 | -37 | 44 |
| 82 | DMN | R | Posterior Cingulate | PC | 11 | -54 | 17 |
| 83 | DMN | R | Superior Temporal Gyrus | STG | 52 | -59 | 36 |
| 84 | DMN | R | Superior Frontal Gyrus | SFG | 23 | 33 | 48 |
| 85 | DMN | L | Superior Frontal Gyrus | SFG | -10 | 39 | 52 |
| 86 | DMN | L | Superior Frontal Gyrus | SFG | -16 | 29 | 53 |
| 87 | DMN | L | Superior Frontal Gyrus | SFG | -35 | 20 | 51 |
| 88 | DMN | R | Middle Frontal Gyrus | MiFG | 22 | 39 | 39 |
| 89 | DMN | R | Superior Frontal Gyrus | SFG | 13 | 55 | 38 |
| 90 | DMN | L | Superior Frontal Gyrus | SFG | -10 | 55 | 39 |
| 91 | DMN | L | Superior Frontal Gyrus | SFG | -20 | 45 | 39 |
| 92 | DMN | R | Medial Frontal Gyrus | MFG | 6 | 54 | 16 |
| 93 | DMN | R | Superior Frontal Gyrus | SFG | 6 | 64 | 22 |
| 94 | DMN | L | Medial Frontal Gyrus | MFG | -7 | 51 | -1 |
| 95 | DMN | R | Medial Frontal Gyrus | MFG | 9 | 54 | 3 |
| 96 | DMN | L | Anterior Cingulate | AC | -3 | 44 | -9 |
| 97 | DMN | R | Anterior Cingulate | AC | 8 | 42 | -5 |
| 98 | DMN | L | Medial Frontal Gyrus | MFG | -11 | 45 | 8 |
| 99 | DMN | L | Medial Frontal Gyrus | MFG | -2 | 38 | 36 |
| 100 | DMN | L | Anterior Cingulate | AC | -3 | 42 | 16 |
| 101 | DMN | L | Superior Frontal Gyrus | SFG | -20 | 64 | 19 |
| 102 | DMN | L | Medial Frontal Gyrus | MFG | -8 | 48 | 23 |
| 103 | DMN | R | Inferior Temporal Gyrus | ITG | 65 | -12 | -19 |
| 104 | DMN | L | Middle Temporal Gyrus | MTG | -56 | -13 | -10 |
| 105 | DMN | L | Middle Temporal Gyrus | MTG | -58 | -30 | -4 |
| 106 | DMN | R | Middle Temporal Gyrus | MTG | 65 | -31 | -9 |
| 107 | DMN | L | Middle Temporal Gyrus | MTG | -68 | -41 | -5 |
| 108 | DMN | R | Superior Frontal Gyrus | SFG | 13 | 30 | 59 |
| 109 | DMN | R | Anterior Cingulate | AC | 12 | 36 | 20 |
| 110 | DMN | R | Sub-Gyral | SG | 52 | -2 | -16 |
| 111 | DMN | L | Parahippocampal Gyrus | PHG | -26 | -40 | -8 |
| 112 | DMN | R | Parahippocampal Gyrus | PHG | 27 | -37 | -13 |
| 113 | DMN | L | Fusiform Gyrus | FG | -34 | -38 | -16 |
| 114 | DMN | R | Pyramis | Pyramis | 28 | -77 | -32 |
| 115 | DMN | R | Middle Temporal Gyrus | MTG | 52 | 7 | -30 |
| 116 | DMN | L | Middle Temporal Gyrus | MTG | -53 | 3 | -27 |
| 117 | DMN | R | Superior Temporal Gyrus | STG | 47 | -50 | 29 |
| 118 | DMN | L | Middle Temporal Gyrus | MTG | -49 | -42 | 1 |
| 119 | DMN | L | Inferior Frontal Gyrus | IFG | -46 | 31 | -13 |
| 120 | DMN | R | Inferior Frontal Gyrus | IFG | 49 | 35 | -12 |
| 121 | VIS | R | Culmen | Culmen | 18 | -47 | -10 |
| 122 | VIS | R | Middle Occipital Gyrus | MOG | 40 | -72 | 14 |
| 123 | VIS | R | Cuneus | Cuneus | 8 | -72 | 11 |
| 124 | VIS | L | Lingual Gyrus | LG | -8 | -81 | 7 |
| 125 | VIS | L | Middle Occipital Gyrus | MOG | -28 | -79 | 19 |
| 126 | VIS | R | Lingual Gyrus | LG | 20 | -66 | 2 |
| 127 | VIS | L | Middle Occipital Gyrus | MOG | -24 | -91 | 19 |
| 128 | VIS | R | Fusiform Gyrus | FG | 27 | -59 | -9 |
| 129 | VIS | L | Declive | Declive | -15 | -72 | -8 |
| 130 | VIS | L | Lingual Gyrus | LG | -18 | -68 | 5 |
| 131 | VIS | R | Fusiform Gyrus | FG | 43 | -78 | -12 |
| 132 | VIS | L | Fusiform Gyrus | FG | -47 | -76 | -10 |
| 133 | VIS | L | Cuneus | Cuneus | -14 | -91 | 31 |
| 134 | VIS | R | Cuneus | Cuneus | 15 | -87 | 37 |
| 135 | VIS | R | Precuneus | Pcu | 29 | -77 | 25 |
| 136 | VIS | R | Lingual Gyrus | LG | 20 | -86 | -2 |
| 137 | VIS | R | Cuneus | Cuneus | 15 | -77 | 31 |
| 138 | VIS | L | Lingual Gyrus | LG | -16 | -52 | -1 |
| 139 | VIS | R | Fusiform Gyrus | FG | 42 | -66 | -8 |
| 140 | VIS | R | Cuneus | Cuneus | 24 | -87 | 24 |
| 141 | VIS | R | Cuneus | Cuneus | 6 | -72 | 24 |
| 142 | VIS | L | Inferior Occipital Gyrus | IOG | -42 | -74 | 0 |
| 143 | VIS | R | Declive | Declive | 26 | -79 | -16 |
| 144 | VIS | L | Precuneus | Pcu | -16 | -77 | 34 |
| 145 | VIS | L | Cuneus | Cuneus | -3 | -81 | 21 |
| 146 | VIS | L | Inferior Occipital Gyrus | IOG | -40 | -88 | -6 |
| 147 | VIS | R | Middle Occipital Gyrus | MOG | 37 | -84 | 13 |
| 148 | VIS | R | Lingual Gyrus | LG | 6 | -81 | 6 |
| 149 | VIS | L | Middle Occipital Gyrus | MOG | -26 | -90 | 3 |
| 150 | VIS | L | Fusiform Gyrus | FG | -33 | -79 | -13 |
| 151 | VIS | R | Inferior Occipital Gyrus | IOG | 37 | -81 | 1 |
| 152 | FPN | L | Precentral Gyrus | PreG | -44 | 2 | 46 |
| 153 | FPN | R | Middle Frontal Gyrus | MiFG | 48 | 25 | 27 |
| 154 | FPN | L | Inferior Frontal Gyrus | IFG | -47 | 11 | 23 |
| 155 | FPN | L | Inferior Parietal Lobule | IPL | -53 | -49 | 43 |
| 156 | FPN | L | Superior Frontal Gyrus | SFG | -23 | 11 | 64 |
| 157 | FPN | R | Inferior Temporal Gyrus | ITG | 58 | -53 | -14 |
| 158 | FPN | R | Medial Frontal Gyrus | MFG | 24 | 45 | -15 |
| 159 | FPN | R | Middle Frontal Gyrus | MiFG | 34 | 54 | -13 |
| 160 | FPN | R | Precentral Gyrus | PreG | 47 | 10 | 33 |
| 161 | FPN | L | Precentral Gyrus | PreG | -41 | 6 | 33 |
| 162 | FPN | L | Middle Frontal Gyrus | MiFG | -42 | 38 | 21 |
| 163 | FPN | R | Middle Frontal Gyrus | MiFG | 38 | 43 | 15 |
| 164 | FPN | R | Inferior Parietal Lobule | IPL | 49 | -42 | 45 |
| 165 | FPN | L | Superior Parietal Lobule | SPL | -28 | -58 | 48 |
| 166 | FPN | R | Inferior Parietal Lobule | IPL | 44 | -53 | 47 |
| 167 | FPN | R | Middle Frontal Gyrus | MiFG | 32 | 14 | 56 |
| 168 | FPN | R | Precuneus | Pcu | 37 | -65 | 40 |
| 169 | FPN | L | Inferior Parietal Lobule | IPL | -42 | -55 | 45 |
| 170 | FPN | R | Precentral Gyrus | PreG | 40 | 18 | 40 |
| 171 | FPN | L | Middle Frontal Gyrus | MiFG | -34 | 55 | 4 |
| 172 | FPN | L | Inferior Frontal Gyrus | IFG | -42 | 45 | -2 |
| 173 | FPN | R | Superior Parietal Lobule | SPL | 33 | -53 | 44 |
| 174 | FPN | R | Inferior Frontal Gyrus | IFG | 43 | 49 | -2 |
| 175 | FPN | L | Middle Frontal Gyrus | MiFG | -42 | 25 | 30 |
| 176 | FPN | L | Medial Frontal Gyrus | MFG | -3 | 26 | 44 |
| 177 | SAN | R | Paracentral Lobule | PCL | 11 | -39 | 50 |
| 178 | SAN | R | Supramarginal Gyrus | SMG | 55 | -45 | 37 |
| 179 | SAN | R | Middle Frontal Gyrus | MiFG | 42 | 0 | 47 |
| 180 | SAN | R | Middle Frontal Gyrus | MiFG | 31 | 33 | 26 |
| 181 | SAN | R | Inferior Frontal Gyrus | IFG | 48 | 22 | 10 |
| 182 | SAN | L | Insula | Insula | -35 | 20 | 0 |
| 183 | SAN | R | Insula | Insula | 36 | 22 | 3 |
| 184 | SAN | R | Inferior Frontal Gyrus | IFG | 37 | 32 | -2 |
| 185 | SAN | R | Claustrum | Cla | 34 | 16 | -8 |
| 186 | SAN | L | Anterior Cingulate | AC | -11 | 26 | 25 |
| 187 | SAN | L | Medial Frontal Gyrus | MFG | -1 | 15 | 44 |
| 188 | SAN | L | Superior Frontal Gyrus | SFG | -28 | 52 | 21 |
| 189 | SAN | L | Cingulate Gyrus | CG | 0 | 30 | 27 |
| 190 | SAN | R | Cingulate Gyrus | CG | 5 | 23 | 37 |
| 191 | SAN | R | Cingulate Gyrus | CG | 10 | 22 | 27 |
| 192 | SAN | R | Middle Frontal Gyrus | MiFG | 31 | 56 | 14 |
| 193 | SAN | R | Superior Frontal Gyrus | SFG | 26 | 50 | 27 |
| 194 | SAN | L | Middle Frontal Gyrus | MiFG | -39 | 51 | 17 |
| 195 | SUB | R | Thalamus | Thalamus | 6 | -24 | 0 |
| 196 | SUB | L | Thalamus | Thalamus | -2 | -13 | 12 |
| 197 | SUB | L | Thalamus | Thalamus | -10 | -18 | 7 |
| 198 | SUB | R | Thalamus | Thalamus | 12 | -17 | 8 |
| 199 | SUB | L | Thalamus | Thalamus | -5 | -28 | -4 |
| 200 | SUB | L | Lentiform Nucleus | LN | -22 | 7 | -5 |
| 201 | SUB | L | Lentiform Nucleus | LN | -15 | 4 | 8 |
| 202 | SUB | R | Lentiform Nucleus | LN | 31 | -14 | 2 |
| 203 | SUB | R | Lentiform Nucleus | LN | 23 | 10 | 1 |
| 204 | SUB | R | Lentiform Nucleus | LN | 29 | 1 | 4 |
| 205 | SUB | L | Lentiform Nucleus | LN | -31 | -11 | 0 |
| 206 | SUB | R | Caudate | Caudate | 15 | 5 | 7 |
| 207 | SUB | R | Thalamus | Thalamus | 9 | -4 | 6 |
| 208 | VAN | L | Medial Frontal Gyrus | MFG | -10 | 11 | 67 |
| 209 | VAN | R | Superior Temporal Gyrus | STG | 54 | -43 | 22 |
| 210 | VAN | L | Superior Temporal Gyrus | STG | -56 | -50 | 10 |
| 211 | VAN | L | Superior Temporal Gyrus | STG | -55 | -40 | 14 |
| 212 | VAN | R | Superior Temporal Gyrus | STG | 52 | -33 | 8 |
| 213 | VAN | R | Superior Temporal Gyrus | STG | 51 | -29 | -4 |
| 214 | VAN | R | Superior Temporal Gyrus | STG | 56 | -46 | 11 |
| 215 | VAN | R | Inferior Frontal Gyrus | IFG | 53 | 33 | 1 |
| 216 | VAN | L | Inferior Frontal Gyrus | IFG | -49 | 25 | -1 |
| 217 | DAN | R | Superior Parietal Lobule | SPL | 10 | -62 | 61 |
| 218 | DAN | L | Middle Temporal Gyrus | MTG | -52 | -63 | 5 |
| 219 | DAN | R | Precuneus | Pcu | 22 | -65 | 48 |
| 220 | DAN | R | Middle Temporal Gyrus | MTG | 46 | -59 | 4 |
| 221 | DAN | R | Superior Parietal Lobule | SPL | 25 | -58 | 60 |
| 222 | DAN | L | Inferior Parietal Lobule | IPL | -33 | -46 | 47 |
| 223 | DAN | L | Precuneus | Pcu | -27 | -71 | 37 |
| 224 | DAN | L | Middle Frontal Gyrus | MiFG | -32 | -1 | 54 |
| 225 | DAN | L | Fusiform Gyrus | FG | -42 | -60 | -9 |
| 226 | DAN | L | Precuneus | Pcu | -17 | -59 | 64 |
| 227 | DAN | R | Precentral Gyrus | PreG | 29 | -5 | 54 |

Note: in the Power atlas, some brain regions are divided into multiple parts, such as the thalamus with three subregions. Some subregions of a certain brain area are classified into different brain functional networks, such as the subregions of precuneus classified into SMN, DMN, VIS, FPN, and DAN, respectively.

Abbreviations: L, left hemisphere; R, right hemisphere; MNI, Montreal Neurological Institute. For the abbreviations of brain networks see Fig. 1.

**Table S6.** The multivariate correlation coefficients (*r*) within TDC and ADHD group for each significant dimension, separately, as well as the statistical between-group differences of *r*.

| Dimension | TDC | ADHD | TDC vs ADHD |
| --- | --- | --- | --- |
| Inattention/hyperactivity  hyperactivity | *r* = 0.615 | *r* = 0.757 | *P* = 0.143 |
| Somatization | *r* = 0.816 | *r* = 0.784 | *P* = 0.632 |
| Inhibition and flexibility | *r* = 0.816 | *r* = 0.855 | *P* = 0.485 |
| Fluency and memory | *r* = 0.894 | *r* = 0.869 | *P* = 0.545 |

Abbreviations: TDC, typically developing children; ADHD, Attention-Deficit/Hyperactivity Disorder.

**Table S7.** The dynamic functional connectivity patterns for each behavioral and cognitive dimension within TDC and ADHD, respectively.

| Dimensions | TDC | |  | ADHD | |
| --- | --- | --- | --- | --- | --- |
|  | loadings of dFC | loadings of clinical variates |  | loadings of dFC | loadings of clinical variates |
| **Inattention/hyperactivity** | | | | | |
|  | SMN-DMN (sum_u = 1.411) | Impulsive-hyperactive behaviors (v = 0.596); Hyperactivity index (v = 0.545); Study problem (v = 0.472) |  | SMN-DMN (sum_u = 1.139) | Study problem (v = 0.846); Impulsive-hyperactive behaviors (v = 0.300) |
|  | DMN-VIS (sum_u = 0.941) |  |  | Within DMN (sum_u = 0.826) |  |
|  | Within DMN (sum_u = 0.692) |  |  | DMN-VIS (sum_u = 0.701) |  |
|  | Within VIS (sum_u = 0.590) |  |  | SMN-VIS (sum_u = 0.682) |  |
|  | DMN-SUB (sum_u = 0.542) |  |  | Within VIS (sum_u = 0.477) |  |
|  | DMN-FPN (sum_u = 0.486) |  |  | SMN-FPN (sum_u = 0.465) |  |
| **Somatization** | | | | | |
|  | SMN-DMN (sum_u = 1.515) | Psychosomatic problems (v = 0.664); Somatic complaints (v = 0.656) |  | DMN-VIS (sum_u = 0.903) | Somatic complaints (v = 0.679); Psychosomatic problems (v = 0.640) |
|  | SMN-VIS (sum_u = 0.939) |  |  | SMN-DMN (sum_u = 0.854) |  |
|  | Within DMN (sum_u = 0.736) |  |  | DMN-FPN (sum_u = 0.827) |  |
|  | DMN-VIS (sum_u = 0.686) |  |  | Within DMN (sum_u = 0.738) |  |
|  | SMN-FPN (sum_u = 0.512) |  |  | SMN-VIS (sum_u = 0.598) |  |
|  | DMN-FPN (sum_u = 0.503) |  |  | DMN-DAN (0.514) |  |
| **Inhibition and flexibility** | | | | | |
|  | DMN-SMN (sum_u = 1.362) | WCST total error (v = -0.384); WCST noperserverative errors (v = -0.381); WCST categories completed (v = 0.353; WCST total correct (v = 0.341); Stroop-C No.right (v = 0.168) |  | DMN-VIS (sum_u = 1.178) | Stroop-C total score (v = 0.422); Stroop-C No. error (v = -0.391); Stroop-C No.right (v = 0.367); WCST categories completed (v = 0.224); WCST total error (v = -0.207) |
|  | DMN-VIS (sum_u = 0.982) |  |  | Within DMN (sum_u = 0.983) |  |
|  | SMN-VIS (sum_u = 0.971) |  |  | DMN-VIS (sum_u = 0.851) |  |
|  | DMN-FPN (sum_u = 0.754) |  |  | DMN-FPN (sum_u = 0.730) |  |
|  | Within DMN (sum_u = 0.700) |  |  | SMN-VIS (sum_u = 0.712) |  |
|  | DMN-SAN (sum_u = 0.534) |  |  | SMN-FPN (sum_u = 0.495) |  |
| **Fluency and memory** | | | | | |
|  | SMN-DMN (sum_u = 1.367) | VM delayed score (v = 0.425); VM immediate score (v = 0.381); VFT word No.error (v = -0.410); VFT word No.correct (v = 0.388); VFT word total score (v = 0.277) |  | DMN-DMN (sum_u = 1.178) | VFT ideational No. correct (v = 0.503); non-VFT No. correct (v = 0.412); VM delayed score (v = 0.280); VM immediate score (v = 0.270); VFT word total score (v = 0.260) |
|  | Within DMN (sum_u = 0.976) |  |  | Within DMN (sum_u = 0.983) |  |
|  | SMN-VIS (sum_u = 0.819) |  |  | DMN-VIS (sum_u = 0.851) |  |
|  | SMN-FPN (sum_u = 0.628) |  |  | DMN-FPN (sum_u = 0.730) |  |
|  | DMN-FPN (sum_u = 0.626) |  |  | SMN-VIS (sum_u = 0.712) |  |
|  | DMN-VIS (sum_u = 0.600) |  |  | SMN-FPN (sum_u = 0.495) |  |

Note: In “loadings of dFC”, we listed the top 10% contributors with the highest sum of absolute loadings within or between networks dFC for each dimension; in “loadings of clinical variates”, we listed all significant behavioral scores (because there were only a few significances) and the top five significant cognitive scores for each dimension if the number of significances was more than five.

Abbreviations: TDC, typically developing children; ADHD, Attention-Deficit/Hyperactivity Disorder; dFC, dynamic functional connectivity; sum_u, sum of absolute u loadings for within- and between-networks. For the abbreviations of clinical measurements and brain networks see Table 1 and Fig. 1.

**4. Supplementary Figures**

**Fig. S1.** The pipeline of participants recruitment. Abbreviation: ADHD, Attention-Deficit/Hyperactivity Disorder.


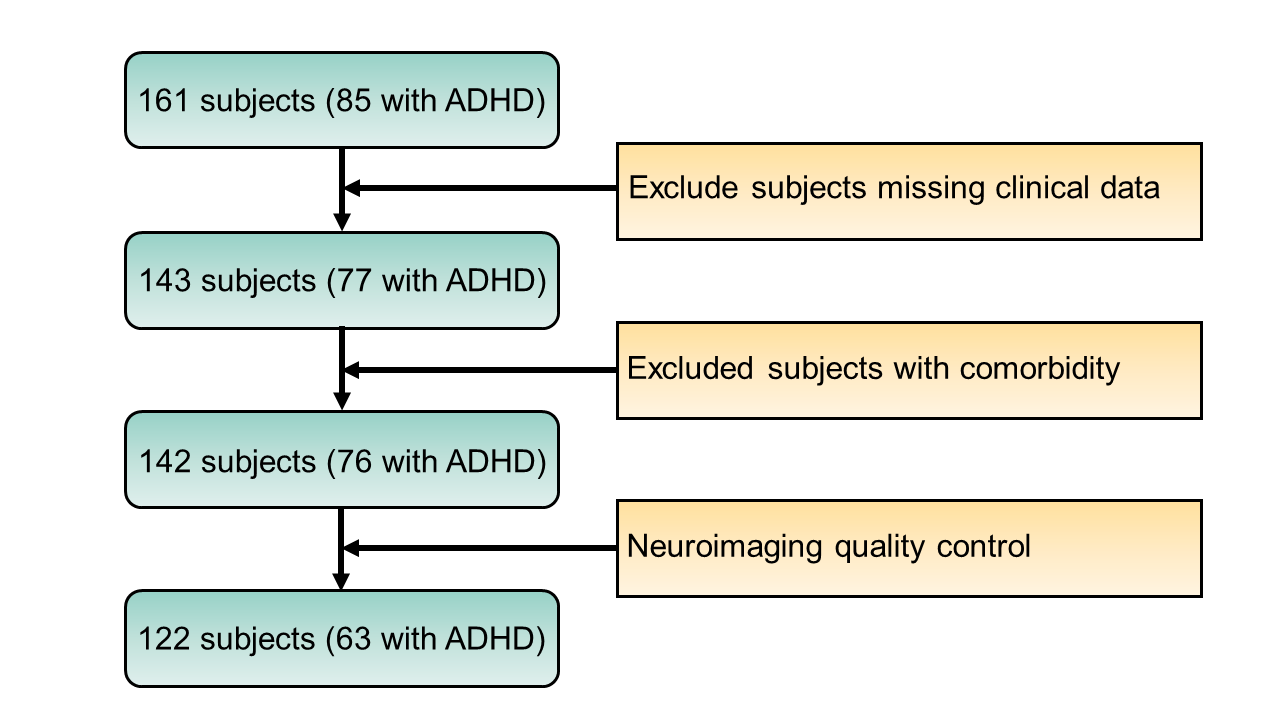


**Fig. S2.** The scatter plots and boxplots show the distribution of behavioral (A) and cognitive (B) scores for typically developing children (TDC) and children with Attention-Deficit/Hyperactivity Disorder (ADHD). See Table 1 for abbreviations of behavioral and cognitive measurements.

**
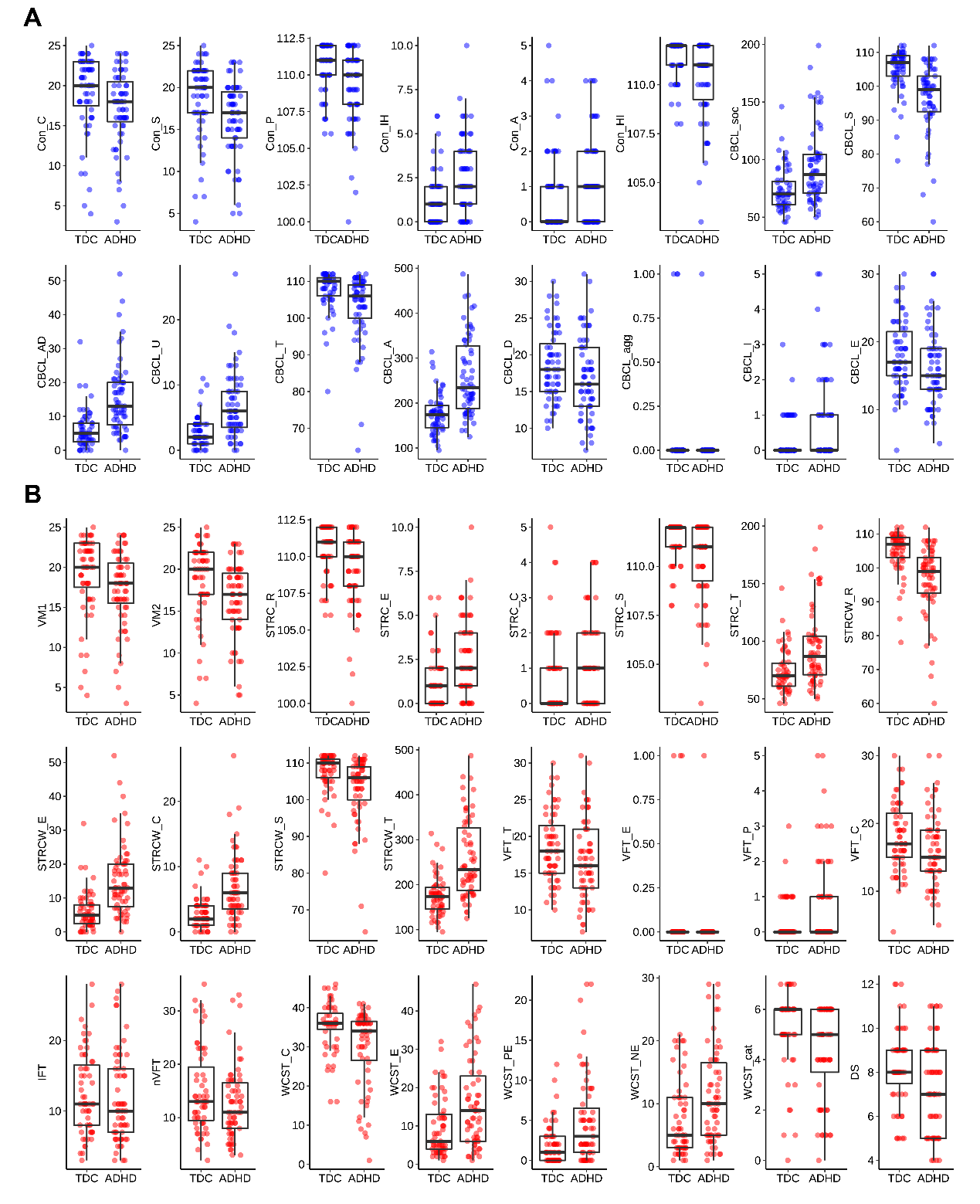
**

**Fig. S3.** The dynamic functional connectivity (dFC) matrices obtained using a sliding-window approach were very similar to those defined using the flexible least squares (FLS) approach.

(A): The mean dFC matrix of all participants was measured by a sliding-window approach. (B): The mean dFC matrix of all participants was measured by FLS method. The warmer color in panels A and B represents higher temporal variability. (C): The similarity between dFC measured by the sliding-window approach and dFC measured by FLS approach. The color of each grid represents the Pearson correlation coefficient between the two measures of dynamics for each pair of brain regions across all participants. The redder color in panel C indicates a higher correlation coefficient. For the abbreviations of brain networks see Fig. 1.


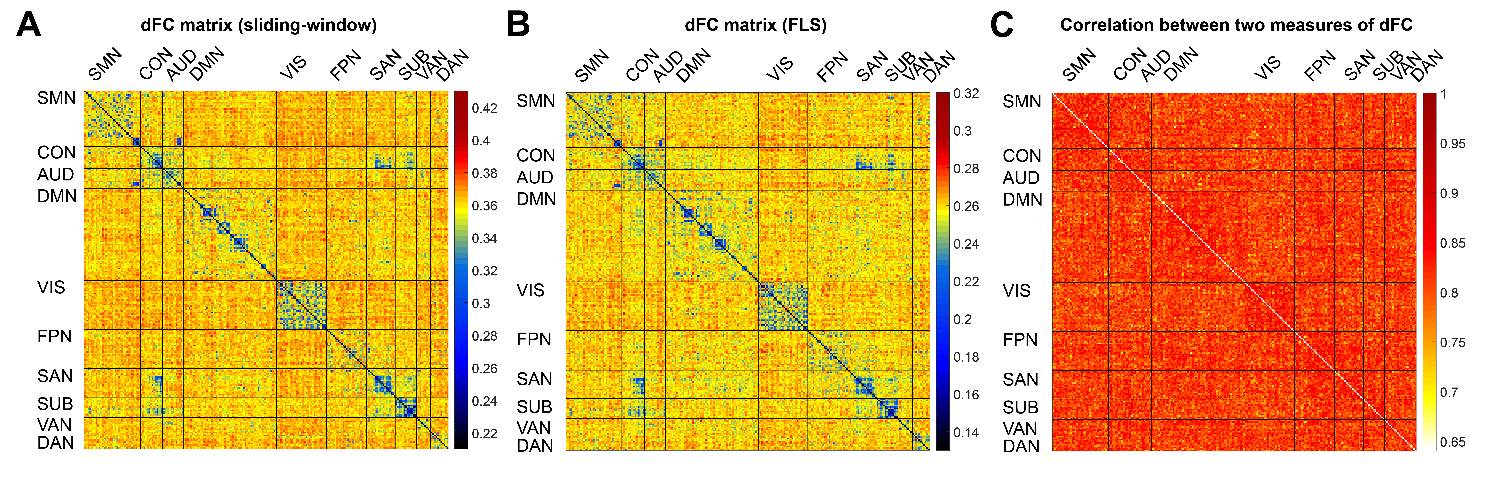


**Fig. S4.** Sparsity parameter search for sparse canonical correlation analysis (sCCA).

We turned the *L*^1^ regulation parameters for the dynamic functional connectivity (dFC) and clinical features in sCCA. The sparsity parameters ranged from 0 to 1, where the higher value indicated the preservation of a larger number of features, and vice versa. (A): Grid search for regularization parameters of dFC-behavior sCCA. (B): Grid search for regularization parameters of dFC-cognition sCCA. The best regularization parameters were (0.7, 0.5) and (0.8, 0.8) for dFC-behavior and dFC-cognition, respectively, shown with red grids.

**
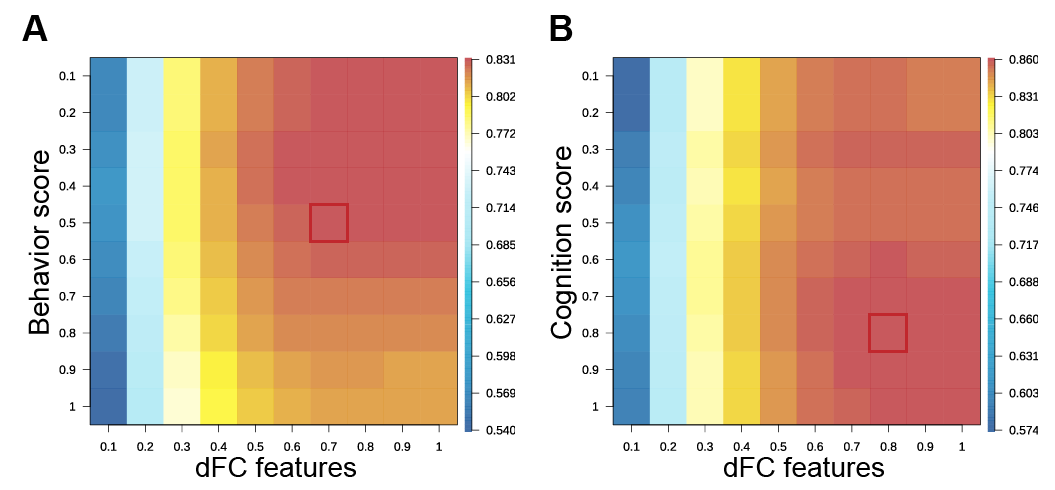
**

**Fig. S5.** Sparse canonical correlation analysis (sCCA) captured multivariate patterns of linked dimensions of dynamic functional connectivity (dFC) patterns and behavior or cognition (which were the main results in the present study).

(A, C): The covariance explained of each canonical variate is shown for dFC-behavior and dFC-cognition sCCA, respectively. The colored dots represented the significant canonical variates (uncorrected *P* < 0.05). (B, D): The canonical correlation coefficients of the significant canonical variates in panels A and C, respectively. Among them, the first two canonical correlations were significant after false discovery rate (FDR) correction. Dashed lines in panels A and C mark the average covariance explained. Error bars in panels B and D denote standard error obtained by the bootstrapping procedure. The ** represents the FDR corrected *P* < 0.05.

**
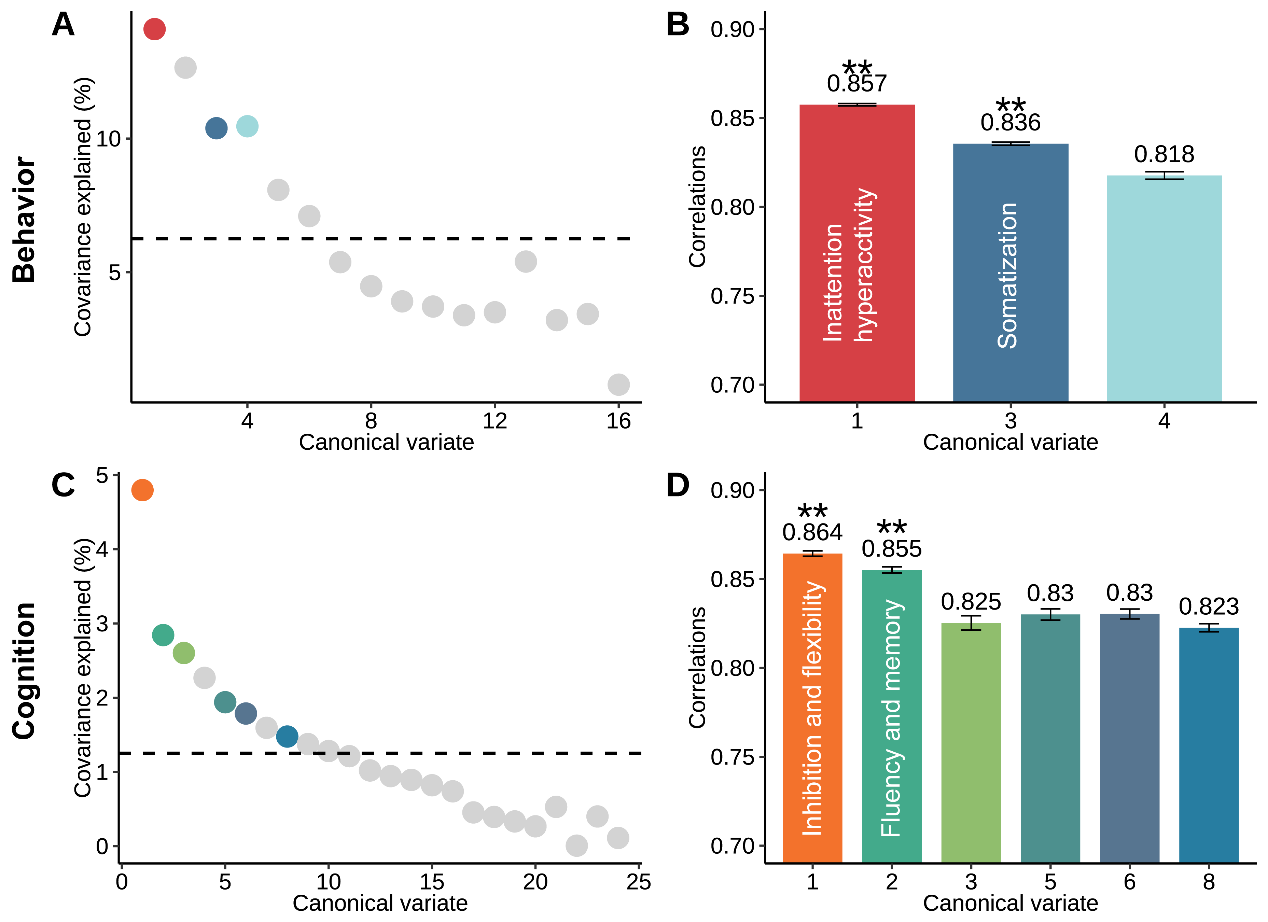
**

**Fig. S6.** Validation analysis: sparse canonical correlation analysis (sCCA) results by using principal component analysis for feature dimension reduction, which was calculated using the sliding-window approach.

The covariance explained by each canonical variate was shown for dynamic functional connectivity (dFC)-behavior (A) and dFC-cognition (C) sCCA, respectively, with the colored dots representing the significant canonical variates (uncorrected *P* < 0.05). The canonical correlation coefficients are shown for two behavior dimensions (inattention hyperactivity and somatization dimensions) (B) and for two cognition dimensions (inhibition and flexibility, fluency and memory) (D), respectively. Dashed lines in panels A and C mark the average covariance explained. Error bars in panels B and D denote standard error obtained by the bootstrapping procedure.

**
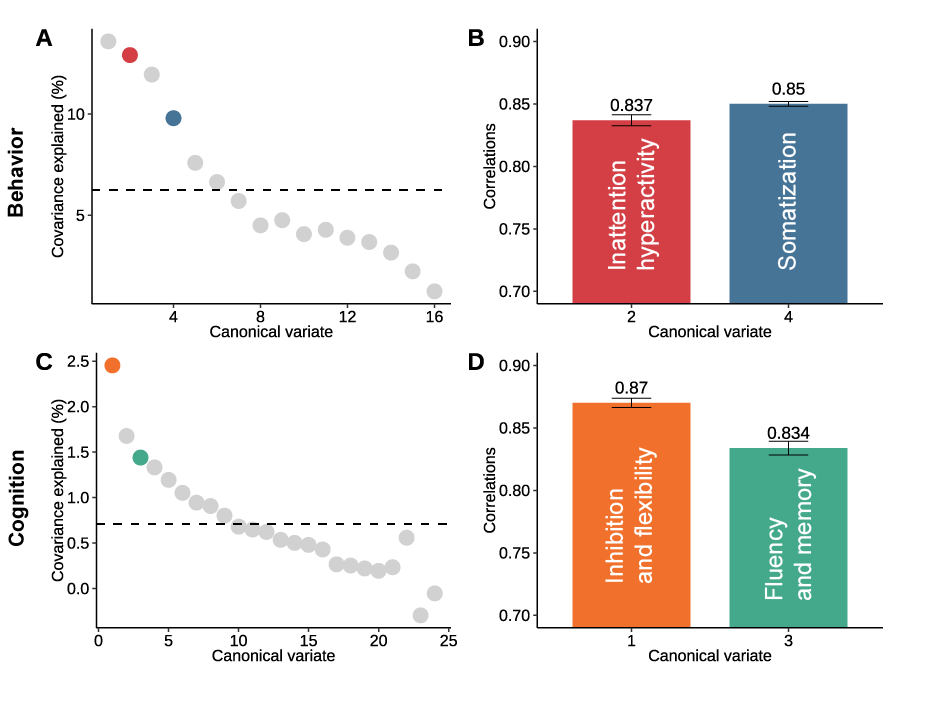
**

**Fig. S7.** Validation analysis: sparse canonical correlation analysis (sCCA) results by using flexible least squares (FLS) method to measure dynamic functional connectivity (dFC) and Relief algorithm for feature dimension reduction.

The covariance explained by each canonical variate was shown for dFC-behavior (A) and dFC-cognition (C) sCCA, respectively, with the colored dots representing the significant canonical variates (uncorrected *P* < 0.05). The canonical correlation coefficients were for two behavior dimensions (inattention hyperactivity and somatization dimensions) (B) and for two cognition dimensions (inhibition and flexibility, fluency and memory) (D), respectively. Dashed lines in panels A and C mark the average covariance explained. Error bars in panels C and D denote standard error obtained by the bootstrapping procedure.

**
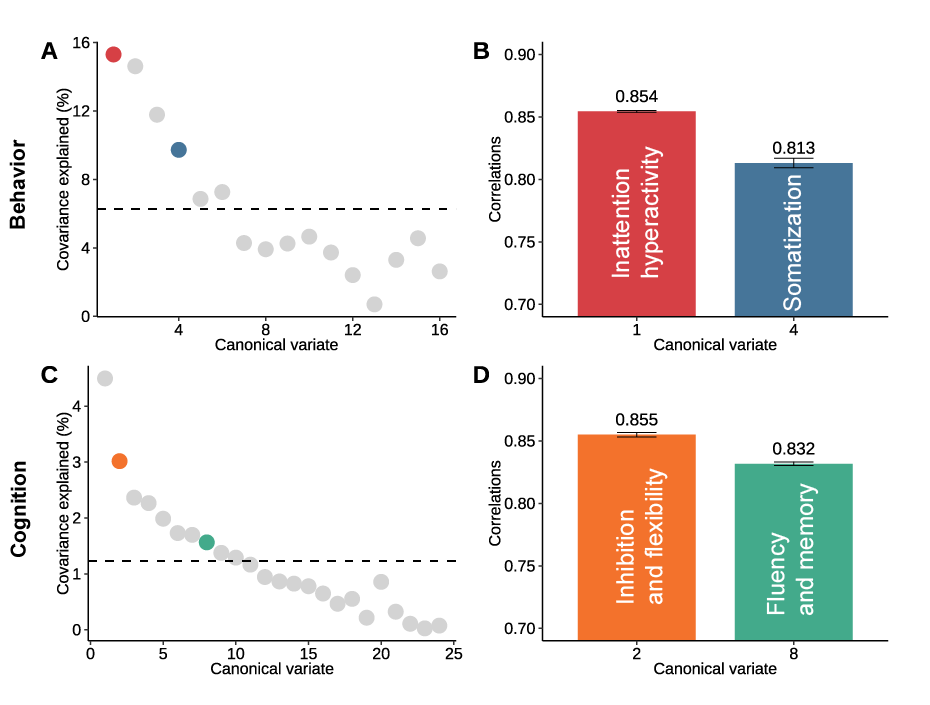
**

**Fig. S8.** A bootstrapping approach was used to identify stable clinical features contributing to each linked dimension.

The resampling distribution was shown for the inattention hyperactivity dimension (A), somatization dimension (B), inhibition and flexibility dimension (C), and fluency and memory dimension (D). The error bar for each clinical feature with a blue dot represents whose 95% confidence intervals (CI) do not cross zero, while error bars with red dots represent whose 95% CI cross zero. For the abbreviations of clinical measurements see Table 1.

**_
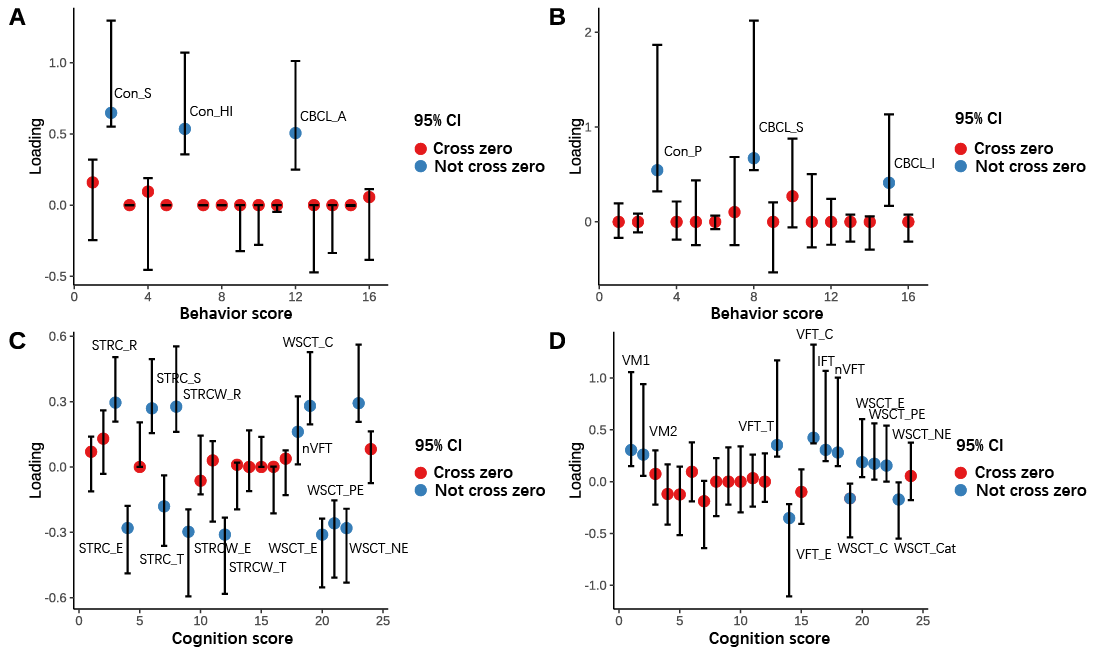
_**

**Fig. S9.** The correlation between each original clinical variable and the corresponding canonical variate in the main results.

The results were shown as the positive correlations between original behavior factor scores and the behavioral canonical variate of the inattention and hyperactivity dimension (A) and the somatization dimension (B), as well as the positive and negative correlations between cognition test scores and cognition canonical variate of the inhibition and flexibility dimension (C) and the fluency and memory dimension (D). For the abbreviations of clinical measurements see Table 1.

**_
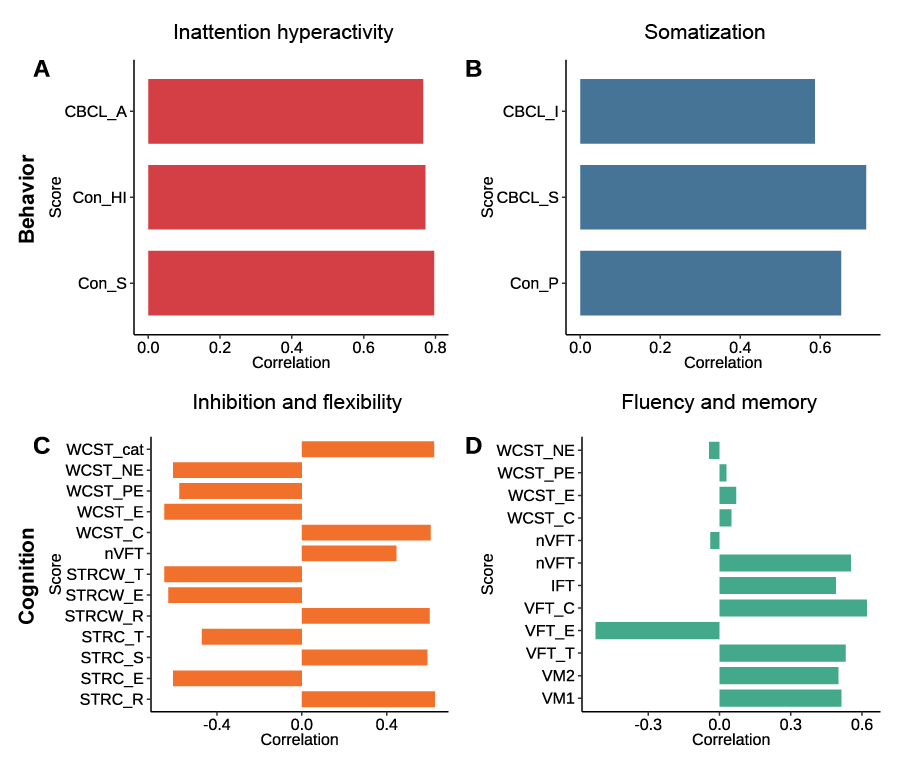
_**

**Fig. S10.** The matrices of pie charts denoting contribution proportion of within- or between-networks dynamic functional connectivity (dFC) with positive and negative loadings, respectively, for each dimension.

The contribution proportion of both positive loadings and negative loadings was shown for the inattention and hyperactivity dimension (A), somatization dimension (B), inhibition and flexibility dimension (C), and fluency and memory dimension (D). The red/blue sector in the pie represents the proportion of positive/negative loadings, respectively. The pies with a red box in each matrix represent the top 10% contributors with the highest sum of absolute loadings for within or between networks dFC for each dimension. For the abbreviations of brain networks see Fig. 1.

**
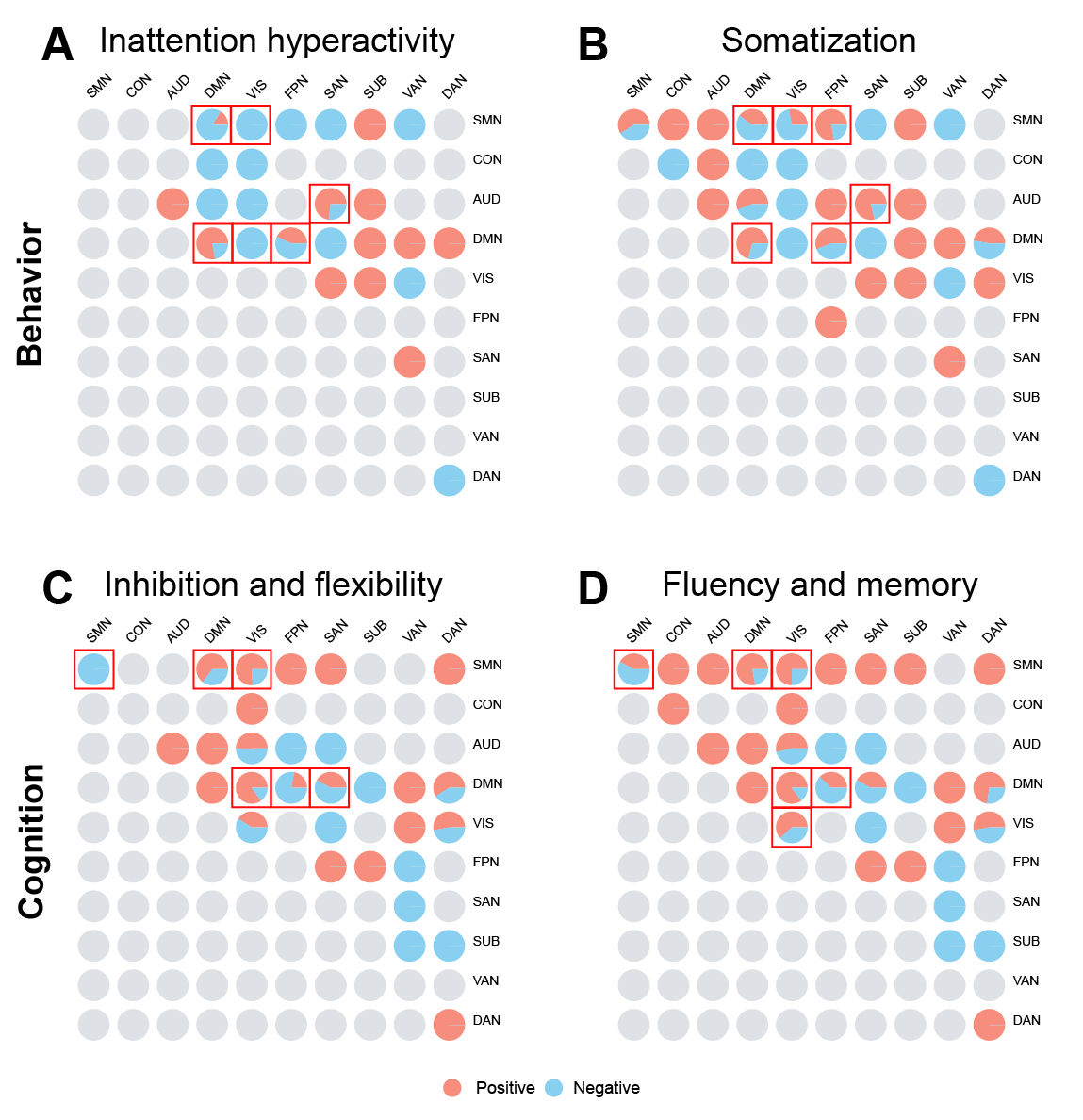
**

**References**

Chai, L. R., Khambhati, A. N., Ciric, R., Moore, T. M., Gur, R. C., Gur, R. E., . . . Bassett, D. S. (2017). Evolution of brain network dynamics in neurodevelopment. *Netw Neurosci, 1*(1), 14-30. doi:10.1162/NETN_a_00001

Kalaba, R., & Tesfatsion, L. (1989). Time-Varying Linear-Regression Via Flexible Least-Squares. *Computers & Mathematics with Applications, 17*(8-9), 1215-1245. doi:Doi 10.1016/0898-1221(89)90091-6

Kira, K., & Rendell, L. A. (1992). *A practical approach to feature selection*. Paper presented at the Proceedings of the ninth international workshop on Machine learning, Aberdeen, Scotland, United Kingdom.

Misic, B., Betzel, R. F., de Reus, M. A., van den Heuvel, M. P., Berman, M. G., McIntosh, A. R., & Sporns, O. (2016). Network-Level Structure-Function Relationships in Human Neocortex. *Cereb Cortex, 26*(7), 3285-3296. doi:10.1093/cercor/bhw089

Sergeant, J. A., Geurts, H., & Oosterlaan, J. (2002). How specific is a deficit of executive functioning for Attention-Deficit/Hyperactivity Disorder? *Behavioural Brain Research, 130*(1-2), 3-28. doi:Pii S0166-4328(01)00430-2

Doi 10.1016/S0166-4328(01)00430-2

Tingley, D., Yamamoto, T., Hirose, K., Keele, L., & Imai, K. (2014). mediation: R Package for Causal Mediation Analysis. *Journal of Statistical Software, 59*(5).

Witten, D. M., Tibshirani, R., & Hastie, T. (2009). A penalized matrix decomposition, with applications to sparse principal components and canonical correlation analysis. *Biostatistics, 10*(3), 515-534. doi:10.1093/biostatistics/kxp008
